# Exploration of a Resequenced Tomato Core Collection for Phenotypic and Genotypic Variation in Plant Growth and Fruit Quality Traits

**DOI:** 10.3390/genes11111278

**Published:** 2020-10-29

**Authors:** Raana Roohanitaziani, Ruud A. de Maagd, Michiel Lammers, Jos Molthoff, Fien Meijer-Dekens, Martijn P. W. van Kaauwen, Richard Finkers, Yury Tikunov, Richard G. F. Visser, Arnaud G. Bovy

**Affiliations:** 1 Plant Breeding, Wageningen University & Research, P.O. Box 386, 6700 AJ Wageningen, The Netherlands; raanatmu@gmail.com (R.R.); jos.molthoff@wur.nl (J.M.); fien.meijer-dekens@wur.nl (F.M.-D.); martijn.vankaauwen@wur.nl (M.P.W.v.K.); richard.finkers@wur.nl (R.F.); yury.tikunov@wur.nl (Y.T.); richard.visser@wur.nl (R.G.F.V.); 2 Graduate School Experimental Plant Sciences, Wageningen University & Research, Droevendaalsesteeg 1, 6708 PB Wageningen, The Netherlands; 3 Bioscience, Wageningen University & Research, P.O. Box 16, 6700 AA Wageningen, The Netherlands; Ruud.deMaagd@wur.nl (R.A.d.M.); Michiel.Lammers@wur.nl (M.L.)

**Keywords:** tomato, *S. lycopersicum*, tomato germplasm, genotyping, phenotyping, domestication, allele mining

## Abstract

A tomato core collection consisting of 122 gene bank accessions, including landraces, old cultivars, and wild relatives, was explored for variation in several plant growth, yield and fruit quality traits. The resequenced accessions were also genotyped with respect to a number of mutations or variations in key genes known to underlie these traits. The yield-related traits fruit number and fruit weight were much higher in cultivated varieties when compared to wild accessions, while, in wild tomato accessions, Brix was higher than in cultivated varieties. Known mutations in fruit size and shape genes could well explain the fruit size variation, and fruit colour variation could be well explained by known mutations in key genes of the carotenoid and flavonoid pathway. The presence and phenotype of several plant architecture affecting mutations, such as *self-pruning* (*sp*), *compound inflorescence* (*s), jointless-2* (*j-2*), and *potato leaf* (*c*) were also confirmed. This study provides valuable phenotypic information on important plant growth- and quality-related traits in this collection. The allelic distribution of known genes that underlie these traits provides insight into the role and importance of these genes in tomato domestication and breeding. This resource can be used to support (precision) breeding strategies for tomato crop improvement.

## 1. Introduction

Wild relatives, old accessions, and landraces held in germplasm collections of crop species represent an underexploited wealth of genetic variation and will, therefore, offer a valuable gene pool to cope with existing and new breeding challenges [1,2]. Among cultivated plants, tomato is in a favourable position, due to the availability of related wild species that can be crossed with cultivated varieties. This has been used in recent years by breeders to diversify their genetic material through trait introgression [1,3,4,5]. Most efforts have focused on the introgression of disease resistance genes and genes that are involved in abiotic stress tolerance, but this gene pool can be used for many other traits as well [1,6,7].

The most notable changes observed during domestication and breeding of tomato concern fruit morphological traits such as fruit size, shape and colour. The molecular basis of these domestication traits has been studied in recent years and several genes affecting these traits have been identified. According to these studies, variation in eight loci has been shown to play a role in transforming the small berries of wild tomatoes to the extremely large fruits that we observe now in modern cultivars [8,9,10,11]. Two loci, *fasciated* (*lcn11.1*, on chromosome 11) and *locule number* (*lcn2.1*, on chromosome 2), have been identified as affecting fruit size by determining the number of carpels in flowers [8,9,11]. According to these studies, *fasciated* and *locule number* affect both the final size and the shape of the fruit. Although *fw11.3* and *lcn11.1* were found to be closely linked and originally thought to represent the same underlying gene or locus [8], cloning of the underlying genes has since then shown that they are distinct loci, with *lcn11.1* renamed *fasciated* [12]. The six other major fruit size loci, *fw1.1, fw2.1, fw2.2, fw3.1, fw3.2,* and *fw11.3*, largely exert their effects on fruit growth and they are able to explain about 67% of total phenotypic variation, resulting in changes in size with little change in shape [11,13]. Similarly, three major loci modulate fruit shape, but with a minimal effect on fruit size. These loci are *ovate* (chromosome 2) [14], *sun* (chromosome 7) [15], and *fs8.1* on chromosome 8 [16]. Both *ovate* and *sun* lead to the formation of elongated or pear-shaped fruits, while *fs8.1* leads to increased fruit length by increasing the cell number in the proximal-distal direction [17]. The diversity in fruit colour in tomato is the result of different mutations found during domestication and crop improvement, such as *yellow-flesh (r)* [18], *tangerine (t)* [19], *green-flesh (gf)* [20], *old gold (og)* [21], and *y (yellow)* [22]. These mutations have been characterised and they reside in genes that are involved in the biosynthesis of carotenoids or flavonoids, or the degradation of chlorophyll (*gf*). 

Up to 500 different tomato accessions have been (re)sequenced [7,23,24], providing an excellent and untapped resource of promising genetic variation. The availability of such a large number of sequenced tomato genomes facilitates the mapping and cloning of important agronomic or domestication traits, through association mapping and using different types of mapping populations. In this study, we explored a tomato core collection that consists of 122 tomato accessions for variation in several plant growth and fruit quality-related traits, to evaluate the potential of this collection for forward genetics studies. In addition, we evaluated 66 sequenced cultivated accessions of the collection for the presence of known mutations or sequence variations in key genes that underlie important domestication and agronomic traits, including inflorescence architecture, fruit pedicel abscission, fruit number, size and shape, fruit colour, and soluble solid content. This information is not only valuable for the selection of genotypes for further forward genetics studies, but also demonstrates how sequenced genomes can be used to efficiently mine for allelic variation in candidate genes of interest. 

## 2. Materials and Methods

### 2.1. Plant Materials

The core collection for this project consisted of 122 tomato accessions (Table 1). Eighty-four of these accessions were selected from the 150-genome (re)sequencing project [7] and consisted of 52 cultivated accessions, including tomato landraces and heirloom varieties of *S. lycopersicum* and *S. lycopersicum* var. *cerasiforme*, which had been selected from the EU–SOL tomato core collection and by the participation of companies involved in this project, as well as 32 accessions comprising wild relatives of tomato. Additionally, 38 additional *S. lycopersicum* accessions from the EU–SOL tomato core collection (including Heinz 1706, the origin of the reference genome) were selected to be included in this panel based on a phylogenetic analysis of 343 tomato accessions (described below), to increase the genetic diversity present in this core collection. After phenotyping these 38 accessions, 14 were selected for further analysis and they have been resequenced (see below).

For greenhouse trials and phenotyping, all self-compatible accessions from the original resequencing collection were selected to be included in this project, including related wild species. Of those, 50 cultivated and 19 wild accessions could be grown, in addition to the 38 newly added accessions that are mentioned above. Three accessions, cv. Ponderosa (RF_006), RF_017, and cv. Snowstorm (RF_203) segregated for fruit colour. The fruits of each colour were phenotyped separately. 

For allele mining, 52 cultivated accessions from the original resequencing collection were used, plus the 14 newly sequenced accessions as mentioned above. 

### 2.2. Description of the Greenhouse Trials

Seeds from all of the accessions were sown in January 2013. Five weeks later, the plants were transplanted to the greenhouse. Plants were grown in two greenhouse compartments as two fully replicated randomised blocks with plots of three plants per accession as experimental units. Each greenhouse contained eight gutters and on each gutter 14 accessions with three plants were planted. All of the plants were self-pollinated by vibration or hand-pollination (in case of wild accessions), and seeds were collected. For the phenotyping trial in spring 2014, seeds that were collected from the tomatoes grown in 2013 were grown in the same setup described for 2013.

### 2.3. Phenotyping

The collection was phenotyped for 11 traits related to architecture, yield and fruit quality. Some traits were analysed in both years, while others were evaluated in one year only. The fruit characteristics fruit number, fruit weight, Brix, fruit firmness, fruit colour, and fruit shape were measured in both 2013 and 2014. The five crop growth-related traits abscission zones of fruit pedicels (AZ category), plant growth rate, the extent of vegetative outgrowth of the inflorescence (VOI), time to flowering, and inflorescence architecture were measured in 2014 only. 

Abscission zones were visually observed and divided in three categories according to their visibility and function as the breaking point for the pedicel at harvest: 1. visible and functional; 2: present and visible but less functional; 3: no visible abscission zone. Inflorescence architecture was visually assessed and classified to five categories 1. simple/fishbone; 2: simple and forked; 3: forked; 4: forked and compound; and, 5: compound. The vegetative outgrowth of the inflorescence of each plant was scored in one of three categories (1: no outgrowth, 3: an outgrowth of leaves; 5: an outgrowth of shoots and leaves). The plant growth rate was measured by the number of days from sowing to reaching the attachment wire (3 m). The time to flowering was measured by counting the number of nodes up to the first inflorescence.

For fruit number, the total number of ripe fruits harvested from trusses 1 to 4 of the three plants of each accession in each compartment was counted. Fruit trusses were not pruned. Fruit yield was determined by summing up the total weight of all fruits harvested (at the ripe stage) from trusses 1 to 4. The total weight of all fruits was divided by the fruit number in order to calculate the value for ‘fruit weight’.

Firmness was measured on freshly harvested ripe fruits and expressed as the average of four measurements per fruit around the mid-height circumference of the fruit using a handheld Fruit Hardness Tester (53215, Turoni, Forlì, Italy). The average firmness of at least four fruits per genotype at time of harvest was calculated for each compartment and each season separately. Total soluble solids (Brix) was measured in freshly harvested ripe fruits and then averaged for at least four fruits (one measurement per fruit) per genotype, using an Atago PR-32α brix meter.

### 2.4. Genotyping and Phylogenetic Analysis

A set of 304 tomato accessions from the EU-SOL core collection was genotyped using the SOLCAP infinium SNP array (7720 SNPs) [25]. The data were deposited at https://doi.org/10.5281/zenodo.2385441. These data were combined with resequencing data of 85 tomato accessions, consisting of 84 resequenced accessions described by Aflitos et al. [7] and of the Heinz reference sequence. In total, 46 of the 85 resequenced accessions were also genotyped using the SOLCAP SNP array. These accessions were used to select a set of in total 5611 SNPs that were reliably scored (>90% identical scores per SNP over 60 samples) in both the SOLCAP array and in the resequencing data analysed by SnpEff v3.4 [26]. This resulted in a combined dataset of 343 tomato genotypes and 5611 SNPs (Additional file 1, Table S4), which was used to construct a neighbour-joining tree (100 bootstraps) using the PAST4.1 software [27]. This tree (Additional file 2, Figure S1) was used to select the core collection of 122 genotypes analysed in this study. Marked in green are the 32 wild accessions and in blue are the 52 cultivated accessions that were selected from the 150-genome (re)sequencing project [7]. Marked in red are 38 additional *S. lycopersicum* accessions selected to be included in this panel in order to increase the genetic diversity present in the core collection.

### 2.5. Sequencing

Alignment and Variant Call Format files of previously resequenced accessions [7] were available locally and they are identical to those deposited at the European Nucleotide Archive (study number: PRJEB5235). Genome resequencing of new accessions was performed, as described previously [7]. Genotyping of accessions for mutations or variants that have previously been characterised (Additional file 1: Table S3) was performed using two approaches. For mutations or variants known to be caused by or correlated with small INDELs or SNPs, VCF files were analysed by SnpEff v3.4 [26], using the iTAG2.3 annotation on the SL2.40 tomato reference genome version to detect or predict sequence variation affecting protein sequence. For the detection of mutations that are caused by larger deletions, (retrotransposon) insertions, or chromosomal rearrangements, read alignments to the SL2.40 reference genome sequences were inspected using the Integrative Genome Viewer (IGV) software [28]. Towards this purpose, reads showing a significantly larger than the average distance between pairs were taken as proof for the presence of a deletion at the previously reported genome position underlying the mutation. Nearby accession reads that were not locally paired, but paired with reads from various other genomic locations, as well as the presence of truncated mapped reads at the site of the previously characterised retrotransposon insertion was taken as evidence for the presence of the insertion in that accession. New resequencing data were deposited at the European Nucleotide Archive (study number PRJEB29506).

## 3. Results

### 3.1. Description of the Core Collection

A phylogenetic tree (Additional file 2: Figure S1) was constructed based on genotypic data of a set of 343 genetically diverse tomato accessions. This was done by combining genotypic data obtained using the SOLCAP infinium SNP array (7720 SNPs [25,29]) and SNP data from the resequenced tomato accessions (Materials and Methods). This phylogenetic tree was used in order to select the core collection of 122 accessions used in this study, consisting of 84 resequenced accessions described by Aflitos et al. [7] (Additional file 1: Table S4 and Additional file 2: Figure S1; 32 wild accessions marked in green and 52 cultivated accessions marked in blue) and 38 accessions which were additionally selected from the phylogenetic tree (Figure S1 and Table S4; marked in red), in order to increase the genetic diversity of the collection. Of the latter, 14 accessions were resequenced. Sequence information of, in total, 66 cultivated accessions, and also the reference genome of Heinz 1706, was used for genotyping for mutations and known variants of interest, as shown in Table 1. Wild accessions were not included in the genotyping activities, since they generally contain many more polymorphisms in the target genes, and it is unclear whether and how these influence the traits under study. In total, 107 accessions (88 cultivated and 19 self-pollinating wild accessions) were grown to maturity and phenotyped. 

Two cultivated accessions failed to grow and, hence, could not be phenotyped. Sequence data used for genotyping were publicly available (52 cultivated accessions from the 150 genome project plus the Heinz 1706 reference sequence) or newly generated as part of this study (14 cultivated accessions), producing a total of 67. This number was very likely too low to have enough statistical power for giving meaningful results or to detect rare alleles in a Genome Wide Association Study (GWAS). Therefore, this was not attempted in the current study.

### 3.2. Phenotyping of the Core Collection

**Figure 1:**
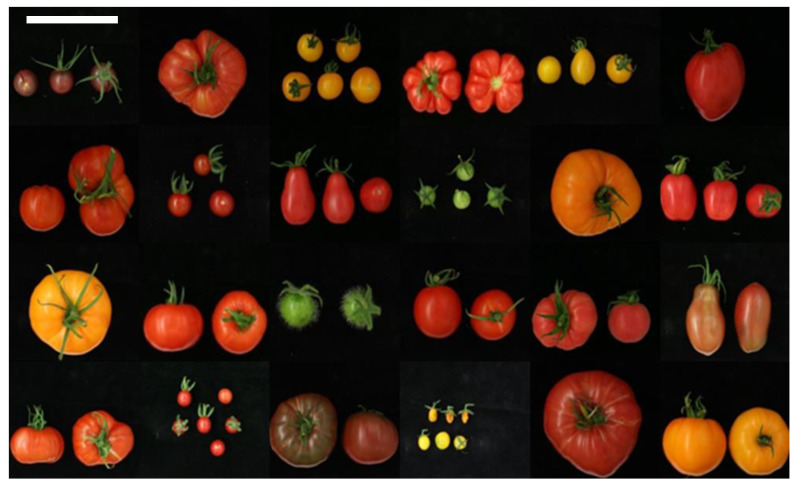
Morphological variation of fruits from the core collection accessions. The picture shows a compilation of fruits from core collection accessions with different colours, shapes, and sizes to illustrate the phenotypic variation present in the core collection. Scale bar is 10 cm.

The set of 107 tomato accessions selected for phenotyping was grown in 2013 and 2014. The plants were phenotyped for architecture, yield, and fruit quality-related traits. The core collection was highly diverse for all the investigated traits, as illustrated for fruit morphology, size, and colour in Figure 1. The results of the observations are shown in Additional file 1: Tables S1 and S2. Table S1 shows the results that were obtained for the crop growth-related traits. These were: abscission zone (AZ), inflorescence branching, the vegetative outgrowth of the inflorescence (VOI), vertical growth rate (days required to reach the crop wire at 3 m), and time to flowering (TtF); the number of nodes up to the first inflorescence). Table S2 shows the results for fruit characteristics, such as the number of ripe fruits harvested per genotype, fruit weight, Brix, firmness, colour, and shape.

### 3.3. Plant Architecture Traits

**Figure 2:**
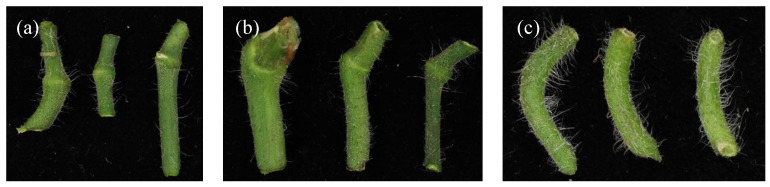
Categories of fruit pedicel abscission zones. Categories were assigned according to their visibility and function as breaking point for the pedicel at harvest. (**a**) 1: Visible and functional; (**b**) 2: Present and visible but less functional; and, (**c**) 3: No visible abscission zone.

The abscission zones (AZ) were scored in three categories, according to their visibility and function as the breaking point for the pedicel at harvest: 1. visible and functional; 2: present and visible but less functional (only breaking with considerable force and breaking more often at the calyx -fruit interface); and, 3: no visible abscission zone (Figure 2). All three AZ categories were found among cultivated accessions: 23 had a clear and functional abscission zone, 59 had a visible, but less functional abscission zone, and only three accessions had no abscission zone at all. All six wild accessions analysed for AZ category had a clearly visible and functional abscission zone, except T495 (RF_043), in which the abscission zone was visible but not functional (Additional file 1: Table S1).

**Figure 3:**
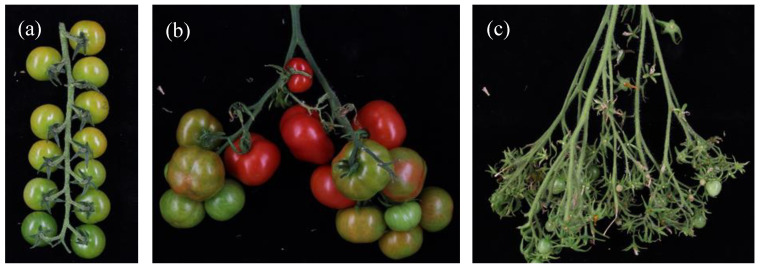
Inflorescence branching categories. (**a**) sSimple or fishbone, (**b**) forked, and (**c**) compound. Based on these three architecture types our genotypes were classified as: 1: simple/fishbone; 2: simple and forked; 3: forked; 4: forked and compound; and, 5: compound.

Three types of Inflorescence architecture were observed in our collection: simple/fishbone, forked, and compound (Figure 3). Most of the genotypes had only one kind of inflorescence architecture, while 21 accessions had two types. Based on these observations, variation in the branching of the inflorescences in our collection was classified as 1. simple/fishbone; 2: simple and forked; 3: forked (one bifurcation); 4: forked and compound; and, 5: compound (two or more subsequent bifurcations). Three cultivated accessions, cv. Katinka Cherry (RF_007), cv. Lidi (RF_014) and DL/67/248 (RF_226) had a compound inflorescence, and this resulted in significantly higher fruit numbers in these accessions (Additional file 1: Tables S1 and S2, Additional file 3: Figure S2). For cultivated accessions belonging to categories 1–4, no relationship was found between fruit number and inflorescence type. All the wild accessions belonged to categories 1, 2, and 3 (Additional file 1: Table S1).

The vegetative outgrowth of the inflorescence (VOI) was scored as categories 1 to 5 (no outgrowth to severe outgrowth) by visual observation of inflorescences. Cultivars Lidi (RF_014), Dana (RF_018) and accession RF_237 showed extreme outgrowth. All of the wild accessions scored from 1–3, while among cultivated accessions all categories were found (Additional file 1: Table S1).

### 3.4. Plant Growth Traits

**Figure 4:**
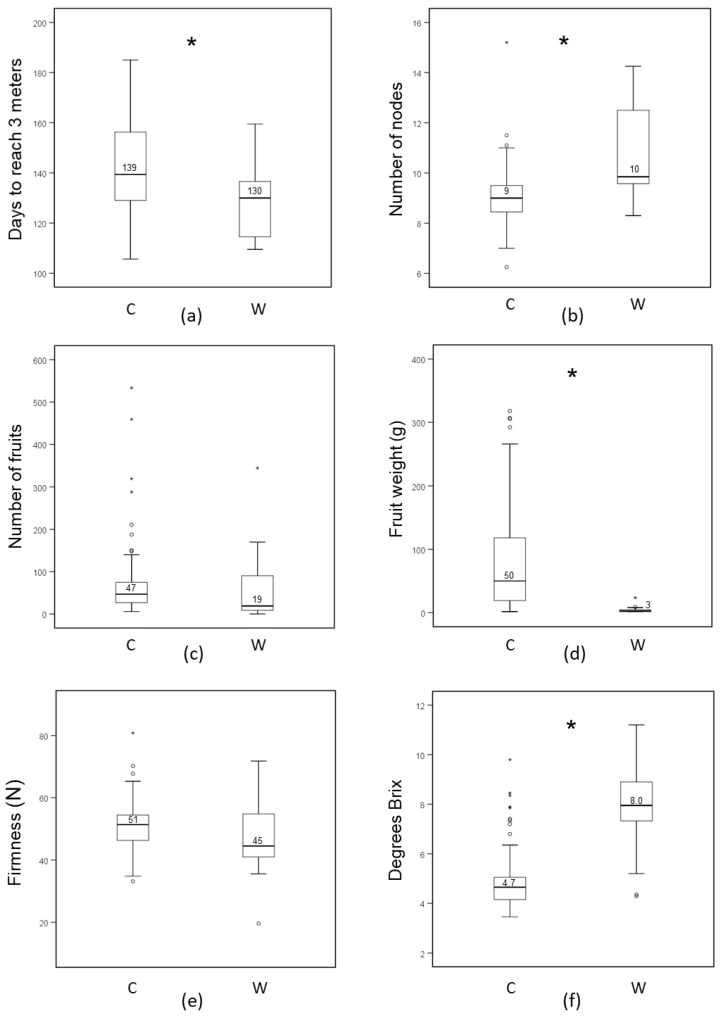
Distribution of fruit yield and fruit quality-related traits among the core collection based on the two classes of genotypes, cultivated (C) and wild (W). (**a**) Plant growth speed; (**b**) Time to flowering; (**c**) Fruit number; (**d**) Fruit weight; (**e**) Fruit firmness; and, (**f**) Degrees Brix. Traits marked with * show a significant difference (*t*-test, *p* < 0.05) between cultivated and wild accessions. Numbers in the plots represent the median value. Outliers are marked with an open circle and far outliers with an asterisk, according to SPSS criteria.

The highest vertical growth rate was observed for tomato accession RF_017 with 97 days to reach the crop attachment wire at three metres and the lowest growth rate was registered for cv. Tessaloniki (RF_096), cv. Rutgers (RF_004) and cv. Jaune Flamme (RF_230) with 185 days, depending on the compartment (Additional file 1: Table S1). A significant correlation was found between the plant growth rate in the two compartments (Additional file 3: Figure S3). The variation in plant growth rate observed for wild and cultivated accessions is shown in Figure 4a. In wild accessions, the plant growth rate is significantly higher than in cultivated tomato, since they need on average fewer days to reach the crop attachment wire (142 days in wild accessions as compared to 129 days in cultivated accessions). 

The number of nodes up to the first inflorescence was counted (Additional file 1: Table S1). The latter varied from six nodes for cv. John’s big orange (RF_008, compartment 1) to 16 for RF_237 (“var. cerasiforme”, compartment 2). On average, flowering started two nodes earlier in cultivated (nine nodes) compared to wild (11 nodes) accessions (Figure 4b). Because the wild accessions grow faster than cultivated accessions (Figure 4a) we cannot exclude that both groups of accessions start flowering at more or less the same time after planting. 

### 3.5. Yield-related Traits

The fruit number varied from less than 10 fruits to more than 500 fruits per plant. Based on the average of both seasons, the highest fruit number was observed for cv. DL/67/248 (RF_226) and cv. Lidi (RF_014), two accessions with compound inflorescences. The lowest fruit number was observed for *S. neorickii* (RF_056) and *S. pennellii* (RF_074) with simple and forked inflorescences, respectively. The variation for fruit number among the collection, based on the average of two seasons, is shown in Figure 4c. Despite the outliers, there was no significant difference in the number of fruits that were produced by cultivated and wild accessions.

**Figure 5:**
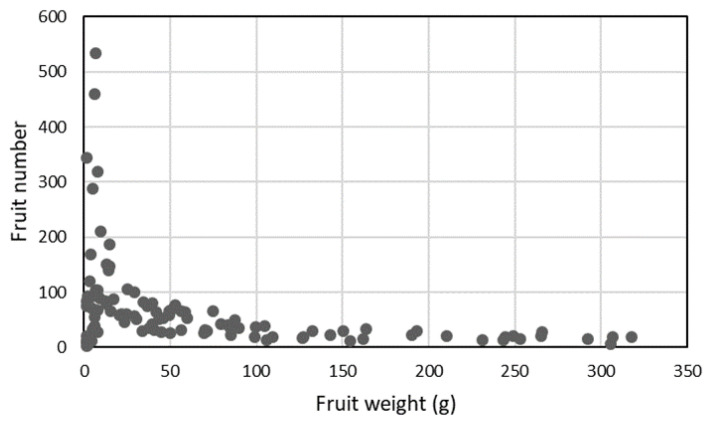
Scatter plot of fruit number versus fruit weight.

**Figure 6:**
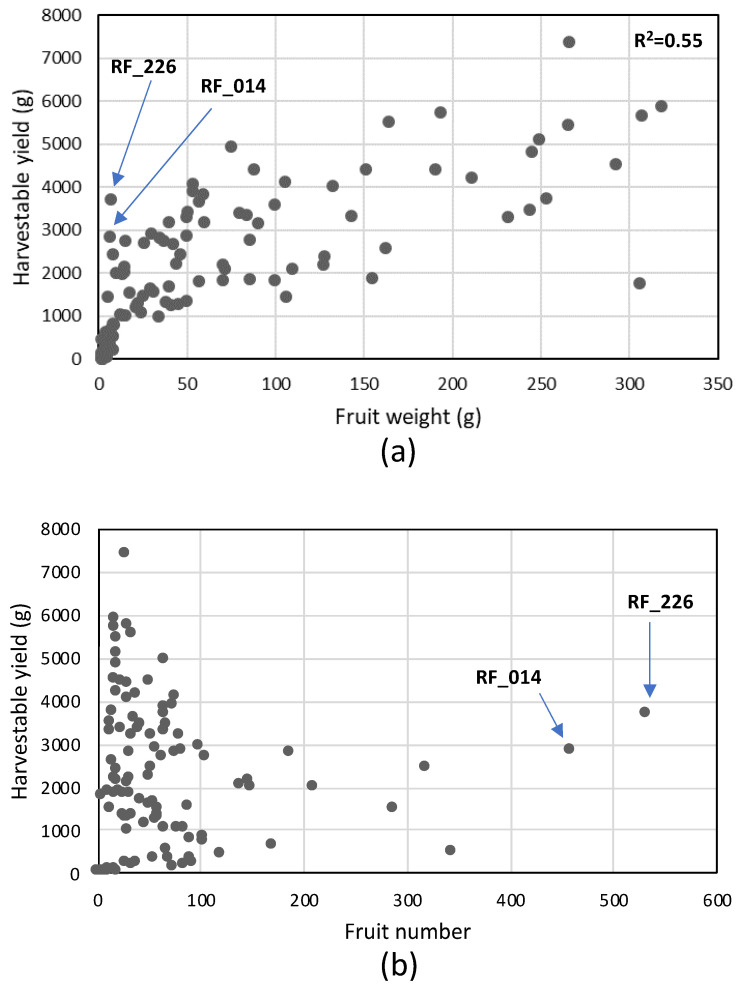
Scatter plot of fruit weight (**a**) and fruit number (**b**) versus harvestable yield.

Fruit weight ranged from 1 g per fruit (N 481-*S. pimpinellifolium* RF_044, 2013) to up to 360 g per fruit (cv. The Dutchman, RF_028, 2014). Fruit weights were highly correlated between the two years (Additional file 3: Figure S4). Cultivated varieties showed a much larger range of variation in fruit weight when compared to wild accessions and their median fruit weight was much higher (50 versus 3 g per fruit, Figure 4d, the average of two seasons). There was a clear negative correlation between fruit number and fruit weight for fruits larger than 10 g, representing cherry and round type tomatoes (Figure 5). This inverse correlation was less evident in very small-fruited, wild accessions in which fruit number varied strongly with little impact on fruit weight. The harvestable yield (total weight of the fruits harvested from the first four trusses of each plant) was predominantly influenced by fruit weight and much less so by fruit number (Figure 6 a,b). Exceptions were the two accessions DL/67/248 (RF_226) and cv. Lidi (RF_014), which had a relatively high yield compared to other genotypes with similar fruit weight, due to their very high fruit number, resulting from their compound inflorescences.

### 3.6. Fruit Quality Traits

The collection harboured accessions with different fruit shapes, such as round, ellipsoid, ovate, rectangular, flat, heart and ox-heart, and varying colours, ranging from pink, yellow, orange, light and dark red, to purple, and striped (Figure 1, Additional file 1: Table S2).

There was extensive variation in firmness among the genotypes in our collection (Additional file 1: Table S2). The highest firmness at harvest in the 2013 season was found for fruits of OH88119 (RF_ 232) and RZ26 (RF_238) with firmness values of 75, and 74.5 Newton (N), respectively, and the lowest firmness was found for the accession var. *cerasiforme* (RF_102) with firmness of 24.4 N. In the 2014 season the highest firmness observed was 80.8 N for the genotypes EZ 033 (RF_231, a reported *rin* mutant), and the lowest firmness was found for *S. neorickii* (RF_057) with firmness of 19.6 N. There was no significant difference in firmness between the groups of cultivated and wild accessions, as shown in Figure 4e. Both groups harbour several accessions with a very high fruit firmness that might potentially be used as novel donors for that trait. Total soluble solids content (Brix) varied from 3.5 (var. *cerasiforme*, RF_103) to 9.8 (tomato, RF_017) degrees in 2013 and from 3.2 (ES 58 Heinz, RF_040) to 11.2 (*S. chmielewskii*, RF_051) degrees in 2014. Brix values correlated well (R^2^ = 0.75) between the two seasons (Additional file 3: Figure S5). Figure 4f shows brix in wild and cultivated accessions (average of two seasons). The box plot reveals that the Brix in most of the wild accessions is significantly higher than in cultivated ones.

**Figure 7:**
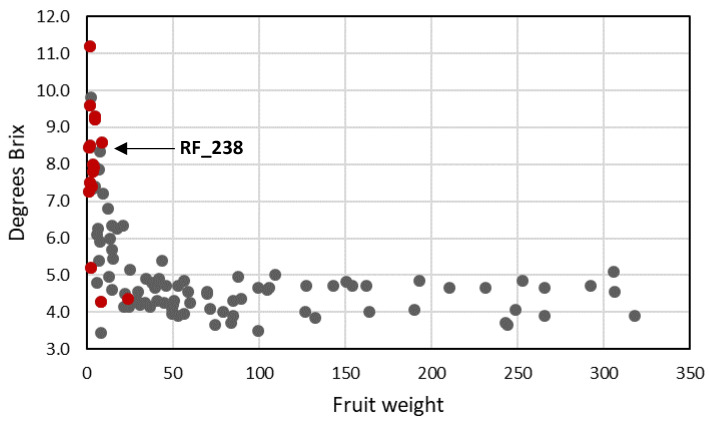
Relationship between Brix (soluble solids content) and fruit weight. Black dots: cultivated tomato accessions; red dots: wild tomato relatives.

We observed an inverse correlation between fruit weight and soluble solids content in accessions with an average fruit weight less than 30 g—cherry type *S. lycopersicum* accessions as well as wild species (Figure 7). These showed an extensive Brix range (from 3 to 10 degrees) and the highest soluble solids levels were found in the smallest fruits. In contrast, fruits with an average weight above 30 g never showed Brix higher than 5.6 degrees in this experiment, but there was no decrease in soluble solids content when fruit weight increased further, in the entire range from 30 to 300 g.

**Figure 8:**
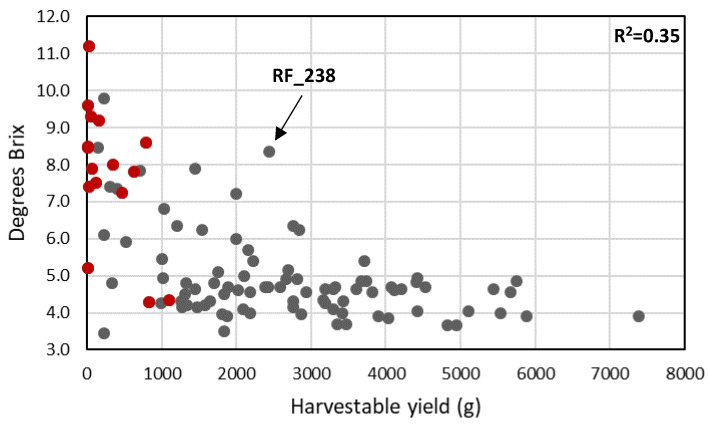
The relationship between Brix (soluble solids content) and harvestable yield. Black dots: cultivated tomato accessions; red dots: wild tomato relatives. The relative outlier RF_238 (see text) is indicated with an arrow.

A similar but less pronounced trend was observed when Brix and harvestable yield were compared (Figure 8): genotypes with high Brix content (higher than seven degrees) had the lowest yield. A notable exception here is cv. RZ26 (RF_238), which combines small fruits and high Brix (8.5) with high fruit number per plant, resulting in a combination of high yield and high Brix. In the lower Brix range, a yield increase without a large penalty on Brix content could be observed. No relationship was found between Brix and fruit firmness (Additional file 3: Figure S6).

### 3.7. Genotyping for Known Mutations or Variants Affecting Plant Architecture, Fruit Size or Shape, or Fruit Colour

All of the resequenced accessions were genotyped for several mutations or variants that are caused by, or strongly linked to known (combinations of) SNPs, small INDELs, or larger deletions, insertions, or rearrangements (Additional file 1: Table S3). SNPs and INDELs were extracted from VCF files, while larger deletions, insertions, or rearrangements were identified by unusual or absent read pairing of accession reads that were mapped to the reference genome. All of the detected mutations or variants in the resequenced accessions are listed in Table 2. For a small number of mutations, the presence in particular accessions was already listed in the Tomato Genomics Resource Centre (TGRC) database or characterised in the literature (as indicated in Additional file 1: Table S3), and their presence was confirmed by genotyping in this study. The accession from which the reference genome was derived, Heinz 1706, itself contains several mutations such as *self-pruning* (*sp*) [30], *uniform* (*u*) [31], and *ovate* (*o*) [32]. Thus, an apparent SNP or INDEL in the majority of other accessions often actually indicates the presence of the wild type or ancestral allele. The classic *sp* allele, leading to a determinate growth habit, is caused by a missense mutation leading to the substitution of a Proline residue at position 76 by a Leucine. This occurs in eleven cultivated accessions (of which three as heterozygous). We identified a likely novel *sp* allele in the determinate accession “Nagcarlan” (RF_227), with a missense mutation leading to the substitution of Glutamine 128 by a Lysine residue. The prediction of the substitution’s effect on protein function using Provean revealed that this substitution with an effect score of -3.7 is likely to be deleterious and thus may well explain the determinate phenotype. Other mutations known to affect plant architecture are rare in our accessions. Two accessions with highly branched inflorescences cv. Lidi (RF_014) and accession DL/67/248 (RF_226) contain the *compound inflorescence* (*s*) [33] mutation affecting the function of a *WUSCHEL* homolog (Additional file 1: Table S1). Four accessions have the previously identified *c* or “potato leaf” mutation as the *c-1* allele, caused by a retrotransposon *Rider* insertion in the third exon of an MYB transcription factor encoding gene (Additional file 3: Figure S7A) [34,35]. A novel mutant allele of *C*, characterised by an approximately 400 base pair deletion in the second exon, was found in cv. Galina (RF_005) (Additional file 3: Figures S7A and S8). The recently characterised *jointless-2* mutation caused by a *Rider* transposon insertion in the first intron of MADS-box protein-encoding gene *MBP21* [36,37] (Additional file 3: Figure S7B) was found in the only four accessions having no visible pedicel abscission zone (AZ score 3): Cal J TM VF (RF_027), OH88119 (RF_232), NCEBR2 (RF_233), and 981136 (RF_234) (Additional file 1: Table S1).

**Figure 9:**
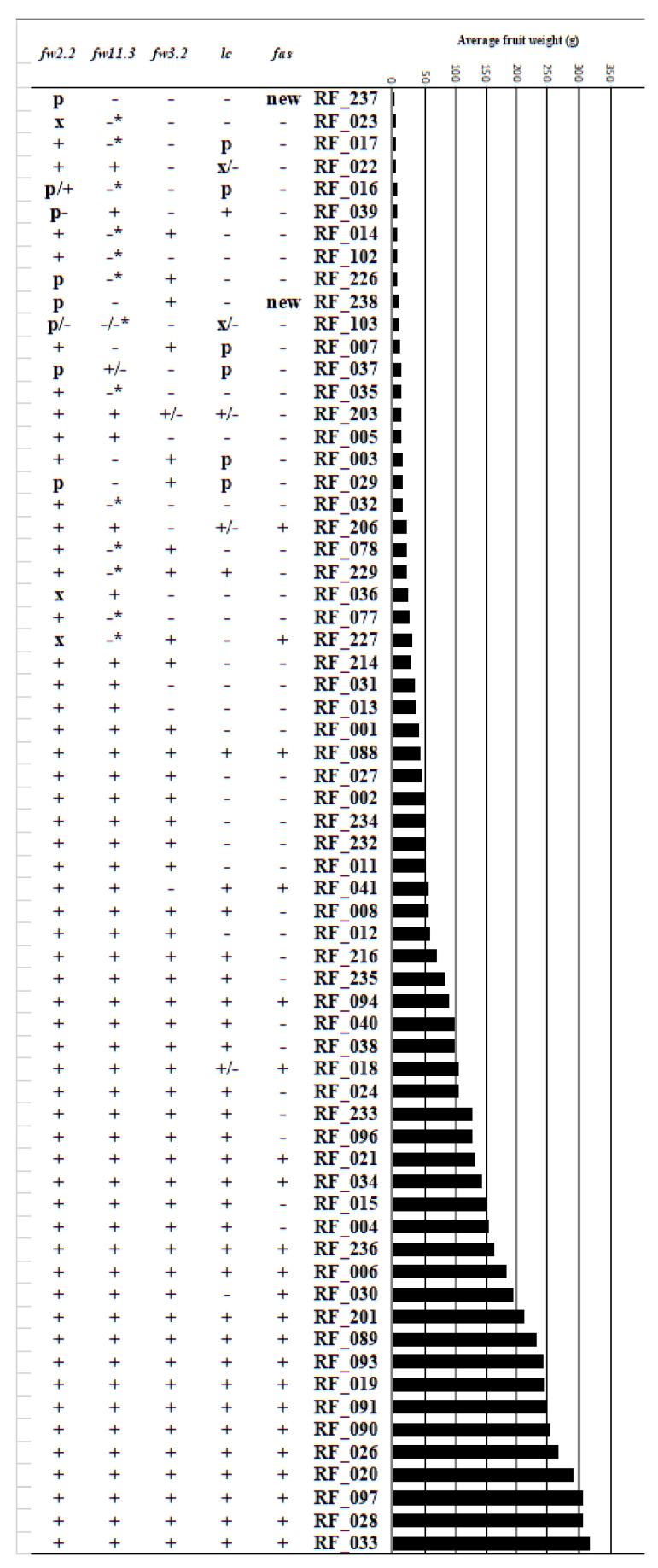
The relationship between size locus haplotype and fruit weight. The different allelic forms are described in the caption of Table 2.

Allelic variation for seven quantitative trait loci (QTLs) for fruit size and weight was investigated by comparing the resequencing data with the published underlying genotypes, as far as they are known so far: *fruit weight 2.2* (*fw2.2*) [38], *fruit weight 3.2* (*fw3.2*) [39], *fruit weight 11.3* (*fw11.3*) [40], *locule number* (*lc*) [13], *fasciated* (*fas*) [41], *ovate* [32], and *sun* [15]. Most cultivated accessions contained the modern, cultivated (large fruit) allele of *fw2.2*. In contrast, a minority consisting of more primitive *S. lycopersicum* accessions contained alleles that were highly similar to those found in our resequenced *S. pimpinellifolium* accessions (Table 2). Two additional accessions contained novel alleles that could not be matched to either the modern cultivated allele or the “*S. pimpinellifolium* allele”. The relationship between size locus haplotype and fruit weight derived from our phenotyping effort is shown in Figure 9. The cultivated accessions with the *S. pimpinellifolium* allele of *fw2.2* have fruits smaller than 10 g. In contrast, the modern, cultivated allele of *fw2.2* is present in all large-fruited accessions (>25 g) in the collection, which is consistent with it being required for large fruit size. Similarly, all of the large-fruited accessions (>25 g) contained the modern large fruit allele of *fw11.3*. For *fw3.2*, cultivated accessions were more equally distributed between having the “wild” or the “modern” (larger fruit) allele. The results of our phenotyping show that all of the fruits with a weight higher than 40 g, with one exception, contain the modern allele of fw3.2 (Figure 9).

**Figure 10:**
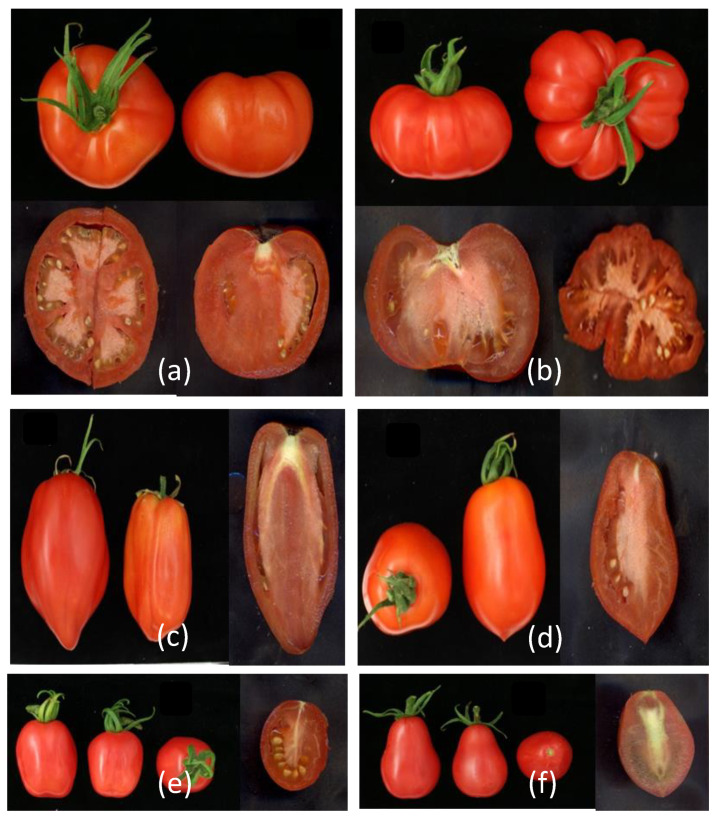
Examples of accessions with fruit shape mutations in our collection. (**a**) RF_096 with the *lc* mutation; (**b**) RF_041 with the *fas* mutation; (c) RF_024 with the *sun* mutation; (**d**) RF_214 with the *ovate* mutation; (**e**) RF_035 with the *ovate* mutation; and, (**f**) RF_043 with the *sun* and *ovate* mutations.

The *locule number* (*lc*) QTL is determined by two SNPs near the tomato *WUSCHEL* ortholog [13], but their effect on *WUS* expression or function has yet to be determined. The large and small fruit alleles of *lc*, respectively, occur throughout the resequenced collection, as also does an allele resembling that of *S. pimpinellifolium* accessions and two unique alleles (Additional file 1: Table S3). Most of the big-fruited cultivated accessions have the modern, large fruit *lc* allele, although this allele is neither a requirement for a big fruit size (e.g., ANTO; RF_030) nor a guarantee (e.g., Morne a L’Eau; RF_229) (Figure 9). The *fas* QTL has been shown to have a greater effect on locule number and fruit size than *lc* [13]. Indeed, the modern, large fruit allele of *fas* is present in the cultivated accessions that have the highest average fruit weights (Figure 9; Additional file 3: Figure S7C). Two mutations that affect fruit shape, *ovate* [32] and *sun* [15], were also investigated. The occurrence of the *ovate* allele, which is also present in the reference accession, was previously reported for most accessions, and is newly reported here only for Nagcarlan (RF_227) and RZ26 (RF_238). The *sun* locus, which causes elongated fruit types, was found in 9 accessions in our collection (Additional file 3: Figure S7D). Figure 10 shows examples of the effect of fruit shape mutations in our collection.

**Figure 11:**
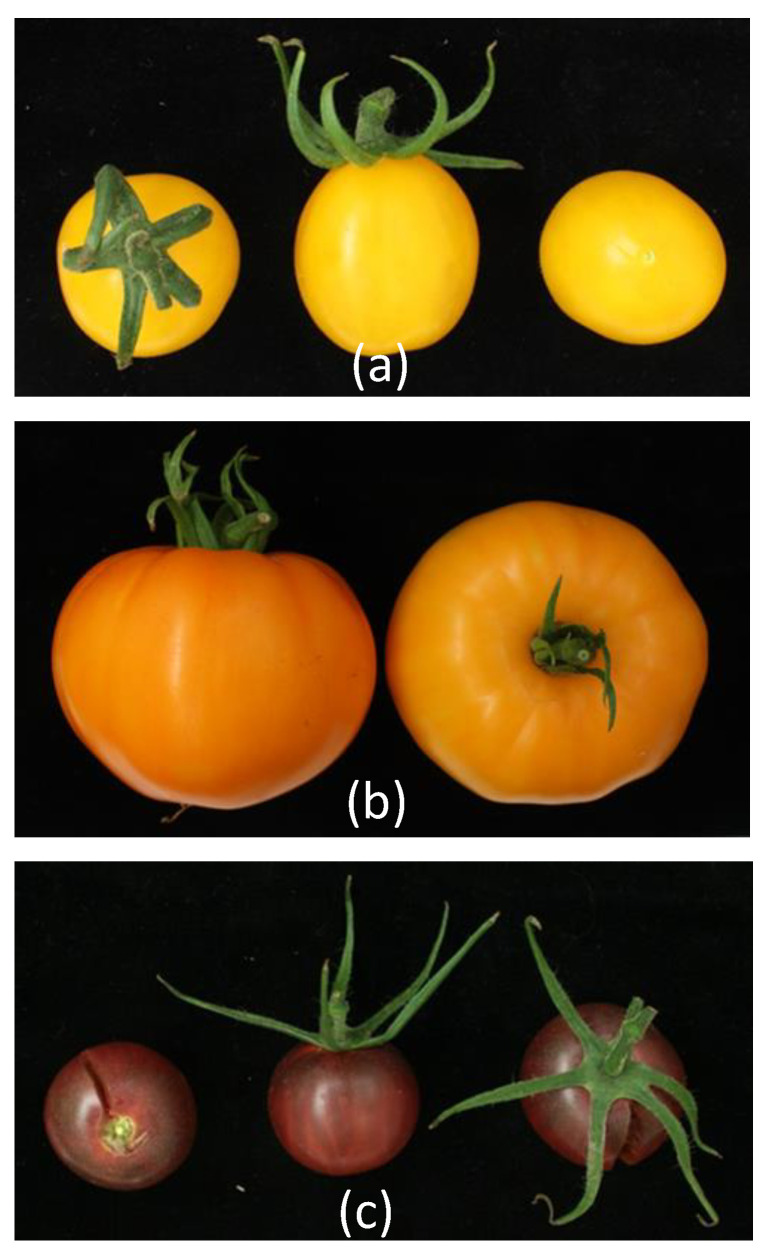
(**a**) RF_032 with the *yellow flesh* mutation; (**b**) RF_006 *Tangerine* mutation; and, (**c**) RF_ 029 with the *old-gold-crimson* and *green flesh* mutations.

Fruit colours in our collection ranged from red through pink to orange or yellow. Moreover, several more modern accessions, including Heinz 1706, contain the *uniform* (*u*) mutation, having a frameshift-causing deletion in the open reading frame of the transcription factor gene *GOLDEN LIKE 2* (*GLK2*). The wild type allele is responsible for the “green shoulder” phenotype of more ancient accessions, such as cv. Ailsa Craig [31]. All of the ripe yellow fruited accessions appeared to contain the *r^y^* allele of the yellow flesh mutation *r*. The latter is detectable as an approximately 6 kb deletion running from the last exon of *PHYTOENE SYNTHASE 1* (*PSY1*) to the first exon of the neighbouring gene [18] (Figure 11a, Additional file 3: Figure S7E). Only one example of the alternative *r* allele was found: cv. Snowstorm (RF_203) was heterozygous for *r* and, hence, this cultivar segregated for red and yellow fruit colour (results not shown). The *r* allele contains an insertion of the retrotransposon *Rider* [18,35]. Another mutation affecting lycopene synthesis, *tangerine*, leads to orange fruits in cv. Ponderosa (RF_006; segregating), cv. Katinka Cherry (RF_007), cv. Dixy Golden Giant (RF_090) and cv. Kentucky Beefsteak (RF_093) and it is caused by a disruption of *CAROTENE ISOMERASE* activity, in this case from the *t^3183^* allele having a deletion located 5’ to the open reading frame [42] (Figure 11b, Additional file 3: Figure S7F). 

The *old-gold-crimson* mutation [43] affecting *LYCOPENE β-CYCLASE* in cv. Black Cherry (RF_029) was reported earlier [7]. The same accession holds the *green flesh* allele *gf^4^* leading to the retention of chlorophyll during ripening, as has been reported earlier [44]. This combination of alleles causes the fruit of this accession to have a deep red, almost black colour at the ripe stage (Figure 11c). Finally, the pink mutation *y* causes pink fruits through the lack of the yellow-coloured naringenin chalcone in the skin of mutant accessions. The latter is caused by deregulated expression of transcription factor gene *MYB12* [45]. Of all sequenced accessions, twenty were homozygous and two were heterozygous for the previously identified *y* allele, having a 603 base pair deletion upstream of the *MYB12* open reading frame as seen earlier in other cultivated accessions [45]. 

There is a single accession, cv. Winter Tipe (RF_031), containing the *non-ripening* (*nor*) mutation [46]. This variety includes a two-nucleotide deletion in the third exon of the transcription factor encoding gene *NAC-NOR*, causing a frameshift mutation and a premature stop codon, which was shown to result in a protein with dominant-negative properties [47]. Another mutation with a strong negative effect on ripening is *ripening inhibitor* (*rin*). EZ 033 (RF_231), is a reported *rin* mutant, which, however, was not resequenced. We did not find this mutation in any of the resequenced accessions and, therefore, it is not reported in Table 2.

## 4. Discussion

In this study, a collection of 122 tomato accessions, including wild relatives, old cultivars, and landraces, was characterised. The collection presented a wide range of phenotypic and genotypic diversity for yield and quality-related traits. This information is expected to be valuable for subsequent tomato breeding programs and crop improvement. In the phenotyping part, we evaluated several traits and compared these traits between wild and cultivated accessions (Figure 4). We observed that there is a clear difference between wild and cultivated accessions for several domestication traits. Cultivated accessions have been selected for higher growth rate, earlier flowering, and production of more and larger fruits when compared to wild accessions.

On the other hand, total soluble solids content was on average higher in wild accessions compared to cultivated accessions. This may be partly due to a dilution of fruit soluble solids with increasing fruit weight or increasing total yield, which might partially explain the inverse correlation between fruit size and Brix in small-fruited accessions (<30 g). Still, this correlation is lacking in bigger fruits (Figure 8). Similar trends, although less apparent, were observed when the total harvestable yield was compared with fruit soluble solids content (Figure 9). This, and the observation of cv. RZ26 with high yield and high Brix, suggests that soluble solids content can be influenced by genetic factors that do not affect total yield (although fruit size of RZ26 is still small). This was also shown for the Brix 9-2-5 QTL, caused by variation in the *LIN5* gene, which was introgressed from *S. pennellii* in a cultivated tomato background and led to a significant increase in Brix without an adverse effect on fruit size and yield [48]. Discovery and stacking of such QTL’s and their underlying alleles offer opportunities for the development of large-fruited tomato genotypes with increased Brix.

Part of the collection, in as far as Aflitos et al. [7] resequenced it, was investigated earlier by Sahu et al. [49], who focused on the analysis of genome-wide sequence variations between wild and cultivated species in order to identify genomic regions and genes that were selected for during domestication. In our study, allele mining was performed for known traits that were related to fruit morphology (shape, colour, and size) and plant architecture. We identified new alleles of the plant-architecture mutations *potato leaf* (*c*) and *self-pruning* (*sp*). Although the accessions carrying these new alleles show the expected phenotype (potato leaves and a determinate growth habit), genetic and/or functional studies are needed in order to demonstrate that these alternative alleles are indeed causal to the observed phenotypes. Leaf shape, as co-determined by *POTATO LEAF*, has recently been shown to determine, or at least be a predictor of, fruit quality and yield [50]. We confirmed the effect of previously identified mutations or alleles for fruit size and shape genes, such as *ORFX* or the tomato ortholog of maize *ZmCNR* (*CELL NUMBER REGULATOR*, *fw2.2*)*, KLUH* (*fw3.2*)*, CSR (CELL SIZE REGULATOR, fw11.3), WUS* (*lc), CLV3* (*CLAVATA3, FAS*)*, SUN,* and *OVATE* in the cultivated germplasm. The genes controlling fruit size and shape can be divided in three categories:(1) the genes that only influence the mass (weight) of the fruit, (2) the genes that influence fruit mass by influencing the locule number, and (3) the genes that only affect the shape of the fruit with no effect on fruit weight. The loci belonging to the first category are *fw1.1, fw2.1, fw2.2, fw3.1, fw3.2,* and *fw11.3*. Lin et al. postulated a two-step model of tomato domestication or improvement from *S. pimpinellifolium* via *S. lycopersicum* cv. *cerasiforme* to modern large *S. lycopersicum* [23]. All five fruit size loci that were cloned and sequenced and could, thus, be used for genotyping our collection (Figure 9) are involved in the second of those steps. Thus, the overall increasing presence of modern alleles of these genes correlated with increasing fruit size can be seen as to represent the evolution and selection from more primitive cv. *cerasiforme* to more modern cultivated tomato. The *fw2.2* locus is considered to be the most critical fruit weight locus so far, and mutation of the underlying gene is considered to be the first step in domestication concerning fruit size [11]. In our collection, most of the cultivated accessions contain the modern (large fruit) allele of *fw2.2*, although many accessions with small fruits do so as well. In contrast, a minority that consists of more primitive *S. lycopersicum* accessions contained alleles that were highly similar to those that were found in our resequenced *S. pimpinellifolium* accessions (Table 2), and these accessions have small fruits. *fw3.2* is another important tomato fruit weight QTL explaining 19% of the fruit mass variance [9]. The *fw3.2* locus also appears to control fruit shape in some tomato varieties and has pleiotropic effects on fruit development [11,51,52]. It was previously believed that the SNP that was associated with the modern allele was somehow responsible for the higher expression of *SlKLUH*, but recently it was shown to be due to the duplication of a chromosome fragment, doubling the number of *KLUH* copies [53]. For *fw3.2*, the “wild” and the “modern” (larger fruit) allele [34] were more or less equally distributed among cultivated accessions. The “modern” large fruit allele of *fw11.3* was found in most of the cultivated accessions in our collection and it is caused by a 1.4 kb 3’ deletion in the *CSR*(Solyc11g071940) gene, leading to a 194 amino acid truncation of the predicted wild-type protein. This allele encodes a partially dominant gain of function protein that affects fruit size by increasing the size of mesocarp cells in the fruit pericarp [35]. *Fw11.3* and *fas* are tightly linked and, therefore, were long thought to be identical. With the cloning and further characterization of the underlying loci or genes in the last few years it has become apparent that although *CSR* (Solyc11g071940) is only approximately 10 kB downstream from the start of the inversion underlying the *fas* allele (Table S3), the two are distinct.

*Fasciated* (*fas*, chromosome 11) [8] and *locule-number* (*lc*, chromosome 2) [13] belong to the second category and they influence the fruit size by controlling the number of carpels in the flower, through the enlargement of the meristem caused by the expansion of the *WUS* expression domain [54]. Wild tomato species, and many cultivated varieties, produce flowers with a gynoecium containing two to four carpels. After fertilization, each carpel develops into a locule in the fruit. Varieties that bear fruit with more locules generally have larger, wider, sometimes ribbed fruits [9]. The large and small fruit alleles of *lc*, respectively, occur throughout the resequenced collection, as does an allele resembling that of *S. pimpinellifolium* accessions and two unique alleles (Table 1). The *fas* QTL has been shown to have a greater effect on locule number and fruit size than *lc* [13]. Although it was originally shown to be linked to two SNPs near a YABBY transcription factor-encoding gene [12], it was more recently shown that the high locule number accessions contain an inversion on chromosome 11. This inversion not only affects *YABBY*, but, more importantly, it also affects the expression of *CLV3* [41].

In our collection, the large fruit alleles of *lc* and *fas* are present in the cultivated accessions with the largest average fruit weights. The genes of category 3 include two mutations that cause a variation in fruit shape with little effect on fruit size, *ovate* and *sun*. The *ovate* mutation is associated with a change from round to elongated or pear-shaped fruit. *OVATE* encodes a protein that belongs to the Ovate Family Protein (OFP) and is thought to, through negative regulation of transcription of target genes, affect cell division patterns in the ovary and ultimately, fruit shape] [32,55]. Recent genetic analyses have further identified *ovate* as a significant quantitative trait (QTL) controlling pear-shaped fruit development in both tomato and eggplant [28]. It has been reported that the mutation is not associated with a single phenotype [9,11], which we also observed in our collection. In some accessions, the *ovate* mutation led to elongated fruit with highly constricted neck growth indicative of pears, but in some backgrounds, neck constriction was not noticeable, and the degree of fruit elongation was not so prominent (Figure 10). Some reports suggested that the *ovate* locus interacts with the *sun* locus on chromosome 7 [11,51]. The *sun* locus is present in nine accessions in our collection. This locus causes elongated fruit types and arose from a *Rider-mediated* transposition event placing an IQ67-Domain (IQD) protein-encoding gene from the ancestral locus on chromosome 10 under the expression control of the *DEFL1* gene on chromosome 7 [15]. Although allelic variation at both *ovate* and *sun* can cause elongated fruit shape, the two loci differ in some important genetic, morphological, and developmental aspects [11].

In this study, we also examined fruit colour and linked our phenotypic observations with known mutations in the carotenoid and flavonoid pigment synthesis pathways. Four yellow accessions in our collection contain the recessive *yellow-flesh* mutation, which is linked to a single locus, *r (red),* on chromosome 3 (Table 2). Locus *r* encodes a fruit-specific phytoene synthase (PSY1), which catalyses the first and rate-limiting step in the carotenoid pathway. Further in the pathway, carotenoid *cis*–*trans* isomerase (CRTISO) produces *all-trans*-lycopene from tetra-*cis*-lycopene (prolycopene) [56,57]. Fruits of tomato with the recessive mutation *tangerine* (*t*) lack this enzyme (due to deletion of 348 bp in the promoter), which leads to the accumulation of tetra-*cis*-lycopene and its precursors upstream in the carotenoid pathway, in particular phytofluene. This results in ripe tomatoes with an orange colour. Twenty-two accessions in our collection contained the previously identified, recessive *y* allele, having a 603 base pair deletion upstream of the *MYB12* gene, encoding a transcription factor, which regulates the accumulation of flavonoids in tomato fruit [45]. Most pink accessions characterised to date harbour this promoter mutation [23], which leads to a ripening-dependent suppression of *MYB12* expression, resulting in a lack of accumulation of the yellow flavonoid naringenin chalcone in the fruit peel and, consequently, a transparent fruit peel and pink appearance of the fruit [45,58]. This phenotype was indeed observed in all 20 accessions with a homozygous *y* allele.

Among all of the accessions in our collection, three accessions (cv. Lidi, DL/67/248 and cv. Katinka Cherry) had highly branched inflorescences with many flowers. Two of these (cv. Lidi and DL/67/248) had the *s* (compound inflorescence) allele. The *S* gene encodes a homolog of the WUSCHEL HOMEOBOX 9 transcription factor and it is involved in regulating inflorescence architecture [33]. Wild tomatoes have a simple inflorescence and branched inflorescences occurred during domestication. Although in some crops (e.g., cereals), selection for a branched inflorescence seems to have been common, in tomato breeding it is rare, presumably due to low fruit set. Yet, our results showed that two accessions with a branched inflorescence produced the largest number of fruits. These had a significantly higher harvestable yield compared to other accessions with the same fruit size. Recent work by others has shown that mutations leading to mild branching can boost yield [36]. All of the wild accessions in our collection had inflorescence architectures belonging to categories 1 (simple), 2 (simple and forked) and 3 (forked), while in the cultivated accessions all five categories were found (Table S1). 

## 5. Conclusions

Current efforts in tomato breeding are focused on broadening the genetic basis of modern tomato, by the introgression of favourable traits from old cultivars, landraces and wild materials. We characterised a diverse collection of sequenced tomato accessions, by phenotypic analysis of important plant growth, yield and fruit quality traits and by genotyping the collection for several mutations or variations in key genes underlying these traits. The results of this study can be used in order to select the most optimal sources for genetic studies of important agronomic and fruit quality traits and crop improvement. 

## Figures and Tables

**Table 1:** Origin and analysis of the accessions that were used in this study.

Origin	Type	Number of Accessions	Phenotyped	Sequenced Genomes for Genotyping
150 genome project	Cultivated	52	50	52
	Wild	32	19	
This Study	Cultivated	38	38	14
Reference (Heinz 1706)	Cultivated			1
	Total	122	107	67

**Table 2:** Detected mutations or variants in the resequenced accessions.

Accession	ID	Name	*fas*	*fw2.2*	*lc*	*SUN*	*fw3.2*	*ovate*	*fw11.3*	*c*	*gf*	*nor*	*og^c^*	*r*	*s*	*sp*	*t^3183^*	*u*	*ug*	*y*	*j-2*
RF_001	LA2706	Moneymaker	-	+	-	-	+	-	+	-	-	-	-	-	-	-	-	+	-	-	-
RF_002	LA2838A	Ailsa Craig	-	+	-	-	+	-	+	-	-	-	-	-	-	-	-	-	-	-	-
RF_003	PI406760	Gardeners Delight	-	+	p	-	+	-	-	-	-	-	-	-	-	-	-	-	-	-	-
RF_004	LA1090	Rutgers	-	+	+	-	+	-	+	-	-	-	-	-	-	-	-	-	-	-	-
RF_005	-	Galina (Galina’s yellow)	-	+	-	-	-	-	+	+	-	-	-	+	-	-	-	-	-	-	-
RF_006	-	Ponderosa	+	+	+	-	+	-	+	-	-	-	-	-	-	-	+/-	-	-	-	-
RF_007	-	Katinka Cherry	-	+	p	-	+	-	-	-	-	-	-	-	-	-	+	+	-	-	-
RF_008	-	John’s big orange	-	+	+	-	+	-	+	-	-	-	-	-	-	+	+	+	-	-	-
RF_011	LA2463	All Round	-	+	-	-	+	-	+	-	-	-	-	-	-	-	-	+	-	-	-
RF_012	LYC 1969	Sonato	-	+	-	-	+	-	+	-	-	-	-	-	-	-	-	+	-	-	-
RF_013	LYC 3897	Cross Country	-	+	-	-	-	+	+	-	-	-	-	-	-	+	-	+	-	-	-
RF_014	LYC 3476	Lidi	-	+	-	-	+	+	-*	-	-	-	-	+	+	-	-	-	-	-	-
RF_015	-	Momatero	-	+	+	-	+	-	+	-	-	-	-	-	-	-	-	-	-	+	-
RF_016	CGN15464	Rote Beere	-	p/+	p	-	-	-	-*	-	-	-	-	-	-	-	-	-	-	-	-
RF_017	LYC 3340	“*Lycopersicon esculentum* Mill.”	-	+	p	-	-	-	-*	-	-	-	-	+/-	-	-	-	-	-	-	-
RF_018	-	DANA	+	+	+/-	-	+	-	+	-	-	-	-	-	-	+	-	+	-	-	-
RF_019	-	Large Pink	+	+	+	+	+	-	+	+	-	-	-	-	-	-	-	-	-	+	-
RF_020	TLYC 3153	“*L. esculentum* Mill.”	+	+	+	-	+	-	+	-	-	-	-	-	-	-	-	-	-	-	-
RF_021	T 828	Bolivar	+	+	+	-	+	-	+	-	-	-	-	-	-	+	-	+	-	-	-
RF_022	PI 129097	“*L. esculentum*”	-	+	x/-	+	-	-	+	-	-	-	-	-	-	-	-	-	-	-	-
RF_023	PI 272654	“*L. esculentum*”	-	x	-	-	-	-	-*	-	-	-	-	-	-	-	-	-	-	+	-
RF_024	-	Jersey Devil	-	+	+	+	+	-	+	-	-	-	-	-	-	-	-	-	-	-	-
RF_026	-	Polish Joe	+	+	+	+	+	-	+	-	-	-	-	-	-	-	-	-	-	+	-
RF_027	CGN20815	Cal J TM VF	-	+	-	-	+	-	+	-	-	-	-	-	-	+	-	+	-	-	+
RF_028	PI 303721	The Dutchman	+	+	+	-	+	-	+	-	-	-	-	-	-	-	-	+	-	+	-
RF_029	LA4451	Black Cherry	-	p	p	-	+	-	-	-	+	-	+	-	-	-	-	-	-	+	-
RF_030	V710092	ANTO	+	+	-	-	+	+	+	-	-	-	-	-	-	-	-	-	+	-	-
RF_031	PC711092	Winter Tipe (nor)	-	+	-	-	-	-	+	-	-	+	-	-	-	-	-	-	-	-	-
RF_032	PI 93302	Chang Li	-	+	-	-	-	+	-*	-	-	-	-	+	-	-	-	-	-	-	-
RF_033	SG 16	Belmonte	+	+	+	+	+	-	+	-	-	-	-	-	-	-	-	-	-	+	-
RF_034	-	Tiffen mennonite	+	+	+	+	+	-	+	+	-	-	-	-	-	-	-	-	-	+	-
RF_035	PI 203232	Wheatley’s Frost Resistant	-	+	-	-	-	+	-*	-	-	-	-	-	-	-	-	-	-	+	-
RF_036	PI 311117	“*L. esculentum*”	-	x	-	+	-	+	+	-	-	-	-	-	-	-	-	-	-	+	-
RF_037	LA1324	“*L. esculentum*”	-	p	p	-	-	-	-/+	-	-	-	-	-	-	-	-	+	-	-	-
RF_038	PI 158760	Chih-Mu-Tao-Se	-	+	+	+	+	-	+	-	-	-	-	-	-	-	-	-	-	+	-
RF_039	LA0113	“*L. esculentum*”	-	p-	+	-	-	-	+	-	-	-	-	-	-	-	-	-	-	-	-
RF_040	LYC 1410	ES 58 Heinz	-	+	+	-	+	-	+	-	-	-	-	-	-	+	-	+	-	-	-
RF_041	PI 169588	Dolmalik	+	+	+	-	-	-	+	-	-	-	-	-	-	-	-	-	-	-	-
RF_042	LYC 2962	Ventura	-	p	p	-	-	-	-	-	-	-	-	-	-	-	-	-	-	-	-
RF_077	-	Large Red Cherry	-	+	-	-	-	-	-*	-	-	-	-	-	-	-	-	-	-	-	-
RF_078	-	Porter	-	+	-	+	+	+	-*	-	-	-	-	-	-	-	-	-	-	+	-
RF_088	-	Bloody Butcher	+	+	+	-	+	-	+	-	-	-	-	-	-	-	-	-	-	-	-
RF_089	-	Brandywine	+	+	+	-	+	-	+	+	-	-	-	-	-	-	-	-	-	+	-
RF_090	-	Dixy Golden Giant	+	+	+	-	+	-	+	+	-	-	-	-	-	-	+	-	-	-	-
RF_091	-	Giant Belgium	+	+	+	-	+	-	+	-	-	-	-	-	-	-	-	-	-	+	-
RF_093	-	Kentucky Beefsteak	+	+	+	-	+	-	+	-	-	-	-	-	-	-	+	-	-	+	-
RF_094	LA1504	Marmande VFA	+	+	+	-	+	-	+	-	-	-	-	-	-	-	-	-	-	-	-
RF_096	-	Thessaloniki	-	+	+	-	+	-	+	-	-	-	-	-	-	-	-	-	-	-	-
RF_097	-	Watermelon Beefsteak	+	+	+	-	+	-	+	-	-	-	-	-	-	-	-	-	-	+	-
RF_102	LA4133	“var. cerasiforme”	-	+	-	-	-	-	-*	-	-	-	-	-	-	-	-	-	-	-	-
RF_103	LA1421	“var. cerasiforme”	-	p/-	x/-	?	-	-	-/-*	-	-	-	-	-	-	-	-	-	-	+/-	-
RF_105	LA1479	“var. cerasiforme”	-*	p	p	-	-	-	-*	-	-	-	-	-	-	-	-	-	-	-	-
RF_201	-	Blondokee	+	+	+	+	+	-	+	-	-	-	-	-	-	-	-	-	-	+	-
RF_203	-	Snowstorm	-	+	+/-	-	+/-	-	+	-	-	-	-	r/-	-	-	-	-	+	+/-	-
RF_206	-	ABC Potato Leaf	+	+	+/-	-	-	-	+	+	-	-	-	-	-	-	-	-	-	-	-
RF_214	LA4345	Heinz 1706 (reference)	-	+	-	-	+	+	+	-	-	-	-	-	-	+	-	+	-	-	-
RF_216	CGN15882	Sonora	-	+	+	-	+	-	+	-	-	-	-	-	-	+	-	+	-	-	-
RF_226	PI 320468	DL/67/248	-	p	-	-	+	-	-*	-	-	-	-	-	+	-	-	-	-	-	-
RF_227	PI 324065	Nagcarlan	+	x	-	-	+	+	-*	-	-	-	-	-	-	+	-	-	-	+	-
RF_229	PI 372385	Morne a L’Eau	-	+	+	-	+	-	-*	-	-	-	-	-	-	-	-	-	-	+	-
RF_232	2K6-6003	OH88119	-	+	-	-	+	-	+	-	-	-	-	-	-	+	-	+	+	-	+
RF_233	2K6-6036	NCEBR2	-	+	+	-	+	-	+	-	-	-	-	-	-	+	-	+	-	-	+
RF_234	2K6-6040	981136	-	+	-	-	+	-	+	-	-	-	-	-	-	+	-	+	-	-	+
RF_235	T 519	Kecskemeti Koria Bibor	-	+	+	-	+	-	+	-	-	-	-	-	-	+	-	+	-	-	-
RF_236	-	Grosse Cotelee	+	+	+	-	+	-	+	-	-	-	-	-	-	-	-	-	-	+	-
RF_237	PI 379007	“var. cerasiforme”	n	p	-	-	-	-	-	-	-	-	-	-	-	-	-	-	-	-	-
RF_238	-	RZ26	n	p	-	-	+	+	-	-	-	-	-	-	-	-	-	-	-	-	-

For *fas* (“-”: absent, as in reference; “+”: present; “n”: new allele). For *fw2.2* (“-”: ancestral allele; “+”: modern allele for large fruit; “p”: *pimpinellifolium*-like allele; “x”: allele of unknown origin). For *lc* (“-”: ancestral allele; “+”: high number allele; “p”: pimpinellifolium allele; “x”: allele of unknown origin). For *sun* (“+”: presence of the duplicated /translocated copy; “-”: ancestral allele). For *fw3.2* (“+”: modern large fruit size allele; “-”: ancestral allele). For *ovate* (“+”: presence of the allele; “-”: absence). For *fw11.3* (“+”: modern allele, as in reference; “-”: wild ancestral allele as in *S. pimpinellifolium* (a 22 nt deletion and large insertion compared to the reference); “-*”: appears to have only the derived 22 nt insertion). For *r* (“+”: *r^y^* allele; “*r*”: alternative mutant *r* allele; “-”: wild type). For all other genes, “+” represents the presence of the mutant allele, and “-” that of the wild type allele.

## Supplementary Materials

The following are available online at https://www.mdpi.com/2073-4425/11/11/1278/s1, Additional file 1: Table S1. Variation among the genotypes for crop growth-related traits. Table S2. Variation among genotypes for fruit characteristics. Table S3. List of mutations and variants screened. Table S4. Genotypic data for 343 genotypes based on 5611 SNPs obtained from SOLCAP and/or resequence data. Additional file 2: Figure S1. Neighbour Joining tree based on 5611 SNP markers and 343 tomato accessions. Additional file 3: Figure S2. Scatter plot of the relationship between inflorescence architecture (categories 1 to 5) and the number of fruits. One-way ANOVA with Post-Hoc Tuckey analysis revealed that there is no statistical difference in fruit number among Inflorescence architecture categories 1 to 4. In contrast, plants with inflorescence type 5 have significantly increased fruit numbers. Figure S3. Scatter plot of plant growth speed between two compartments. Figure S4. Scatter plot of fruit weight between 2013 and 2014 seasons. Figure S5. Scatter plot of Brix content between 2013 and 2014 seasons. Figure S6. Relationship between fruit Brix content and fruit Firmness. Figure S7. IGV output of read alignments demonstrating the presence and nature of structural variations leading to mutations: A. *potato leaf* (*c*). B. *jointless-2* (*j-2*). C. *fasciated* (*fas*). D. *sun*. E. *yellow flesh* (*r^y^*). F. *tangerine* (*t*). Figure S8. Potato leaf mutant allele (c) in cv. Galina (RF_005).
