# Supplementary material for: Exploration of a Resequenced Tomato Core Collection for Phenotypic and Genotypic Variation in Plant Growth and Fruit Quality Traits
Source: Genes (Basel). 2020 Oct 29;11(11):1278. doi: 10.3390/genes11111278 (PMC7692805; doi:10.3390/genes11111278)

Figure S1. Neighbour joining tree (bootstrap n=100) based on 5611 markers and 343 tomato accessions. The 84 accessions sequenced in the 150+ tomato genome project are marked in green (32 wild accessions and blue (52 cultivated accessions). The 38 cultivated accessions additionally selected from this tree are shown in red. The remaining 221 accessions are part of the EU-SOL core collection.

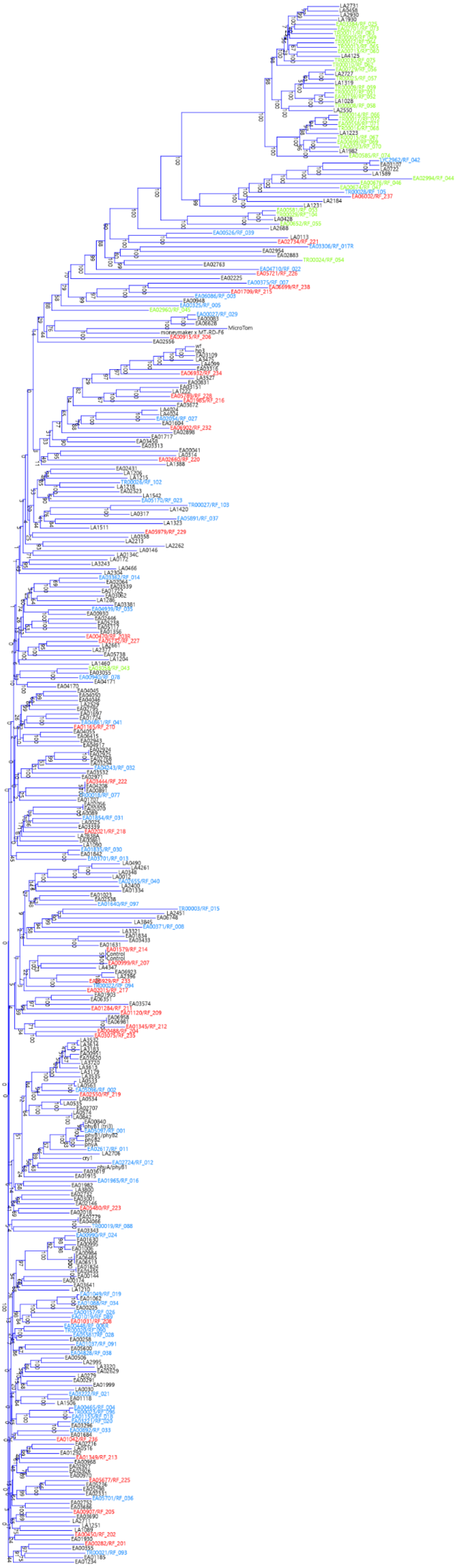

Supplement: Supplementary file 1 [file genes-11-01278-s001.zip › genes-977597-suppl/additional file 2.pdf]
