# Supplementary material for: Exploration of a Resequenced Tomato Core Collection for Phenotypic and Genotypic Variation in Plant Growth and Fruit Quality Traits
Source: Genes (Basel). 2020 Oct 29;11(11):1278. doi: 10.3390/genes11111278 (PMC7692805; doi:10.3390/genes11111278)
Supplement: Supplementary file 1 [file genes-11-01278-s001.zip › genes-977597-suppl/additional file 3.pdf]

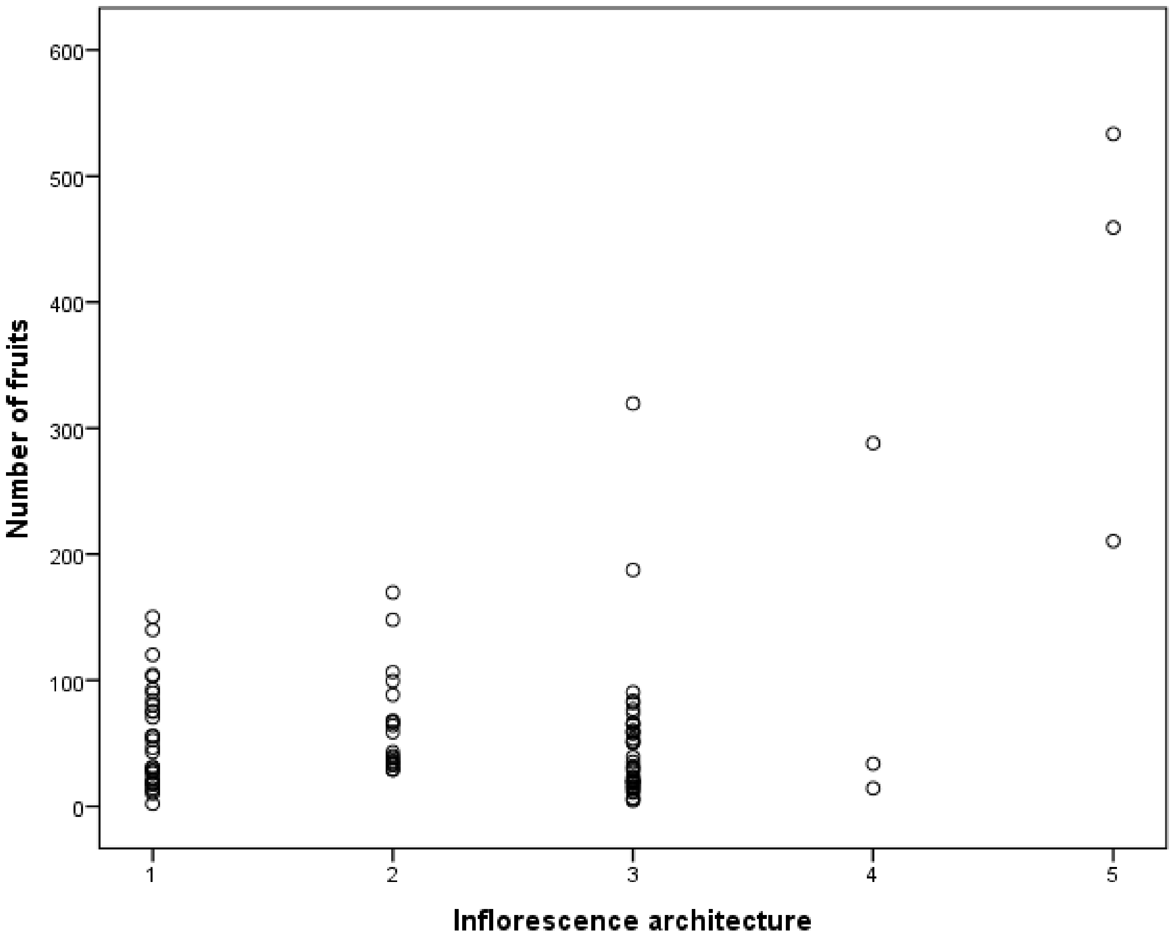

Figure S2. Scatter plot of the relationship between inflorescence architecture (categories 1 to 5) and number of fruits. One-way ANOVA with Post Hoc Tuckey analysis revealed that there is no statistical difference in fruit number among Inflorescence architecture categories 1 to 4, while plants with inflorescence type 5 have significantly increased fruit numbers.

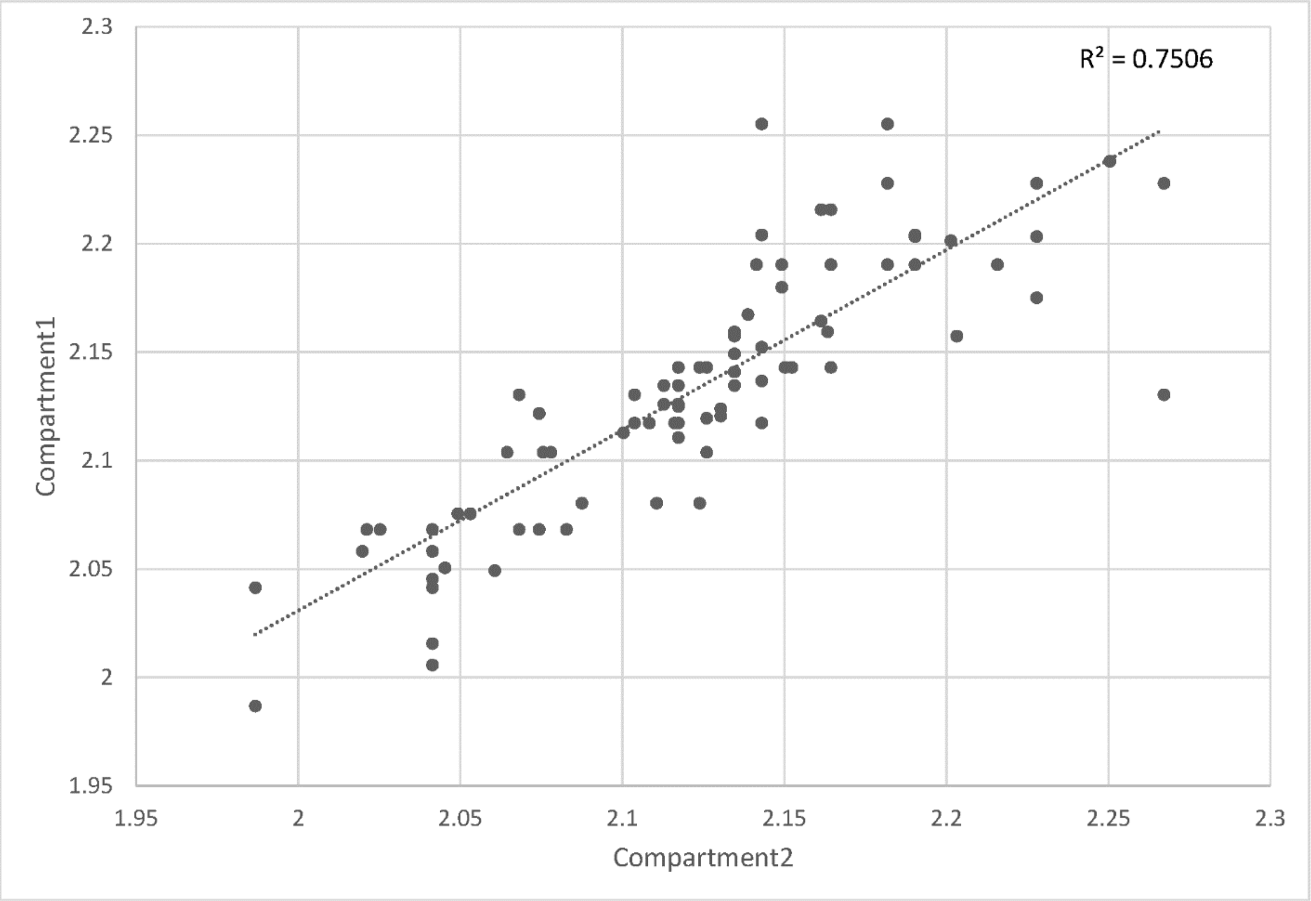

Figure S3. Scatter plot of plant growth speed between two compartments. Numbers indicate the days until the plants reach the crop attachment wire. Values have been transformed to Log scale (base 10).

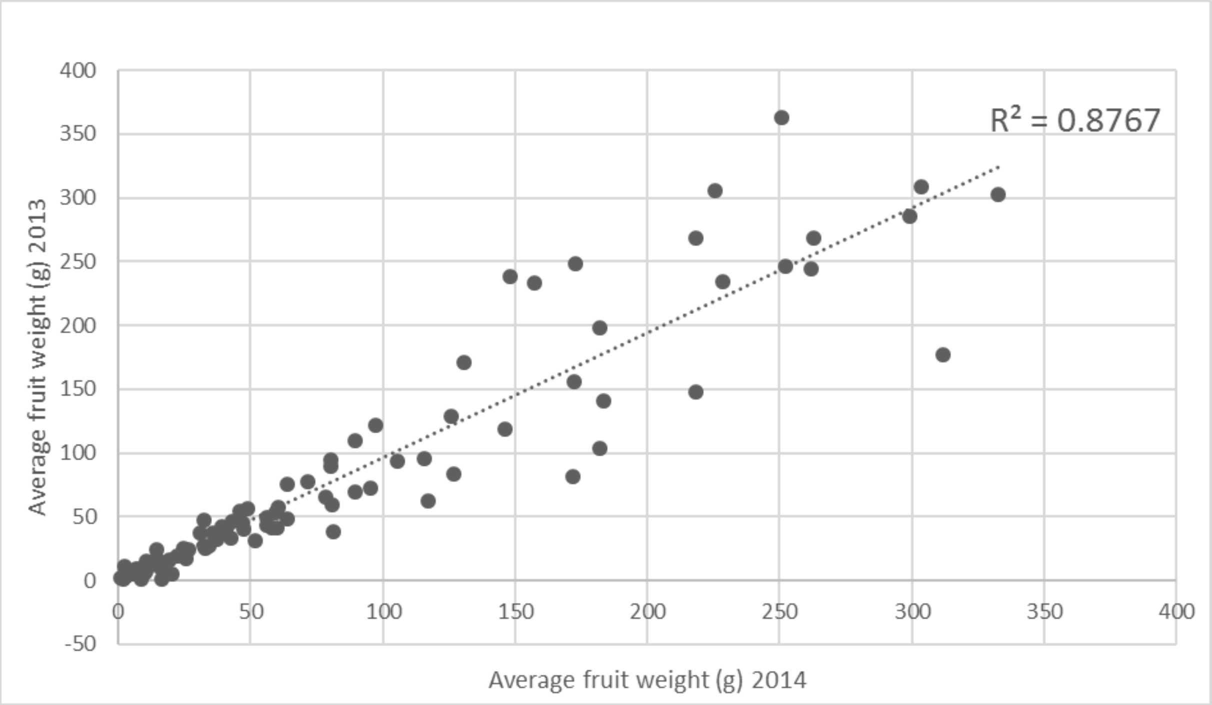

Figure S4. Scatter plot of fruit weight (g) between 2013 and 2014 seasons.

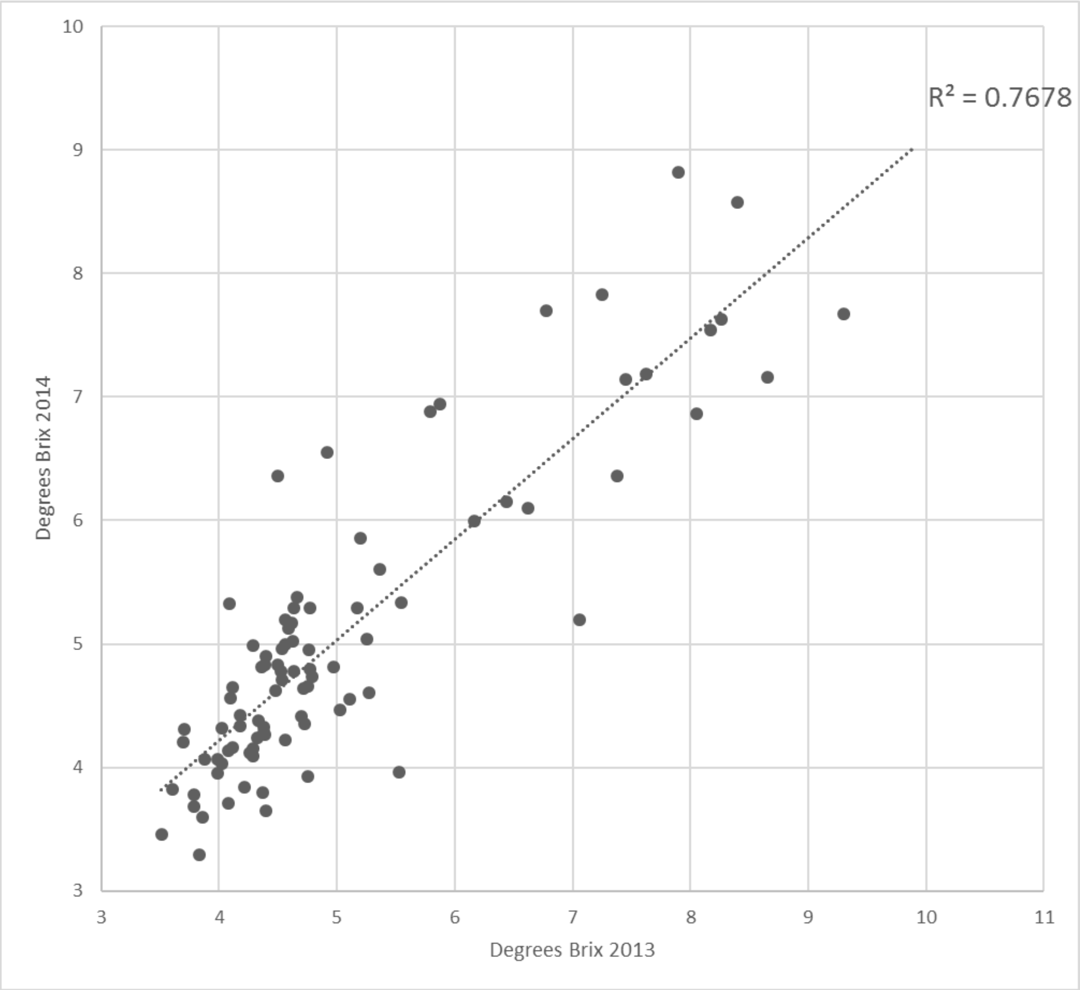

Figure S5. Scatter plot of Brix content between 2013 and 2014 seasons.

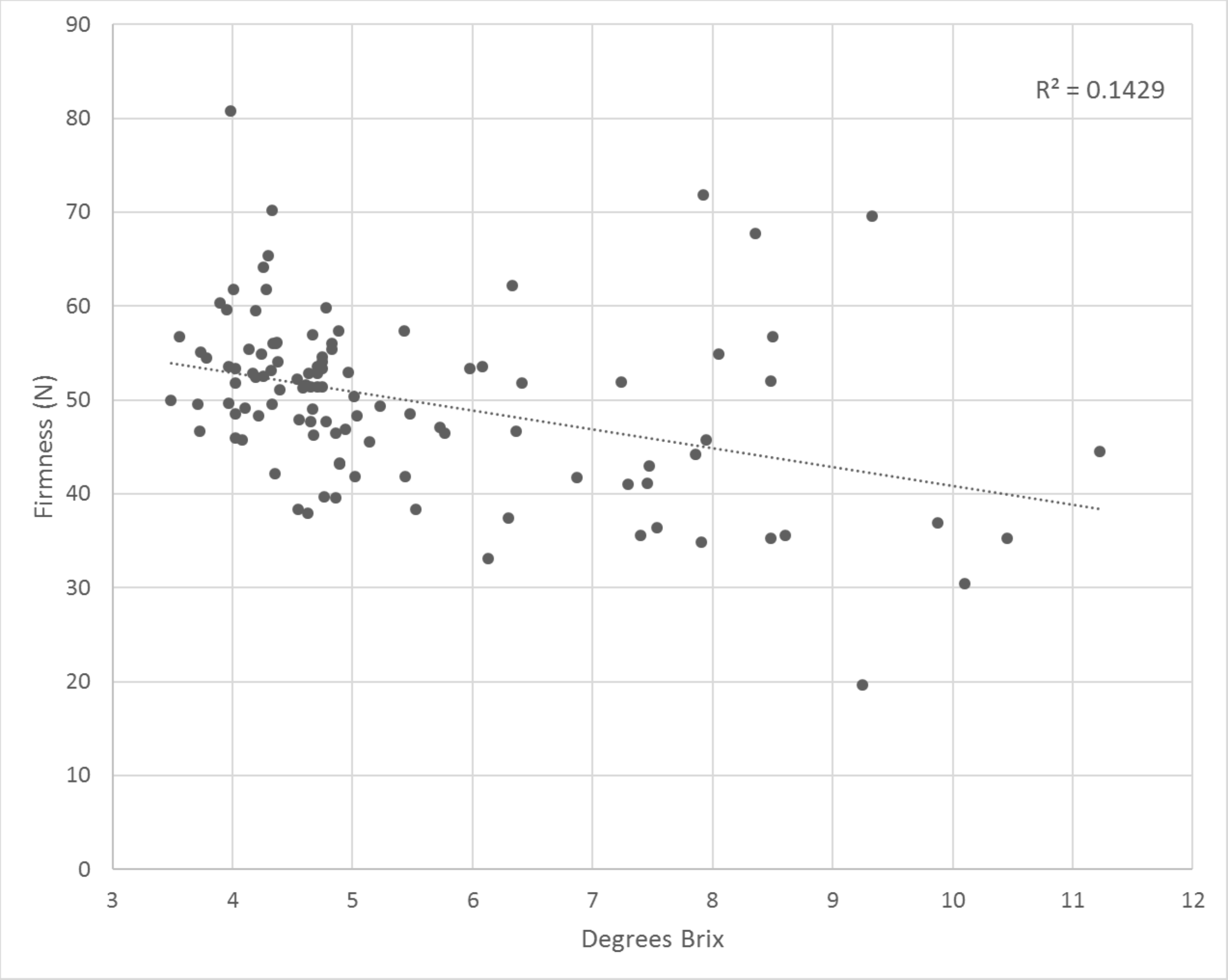

Figure S6. Relationship between fruit Brix and fruit Firmness.

Figure S7. IGV output of read alignments demonstrating the presence and nature of structural variations leading to mutations: **A.** *potato leaf* (*c*). **B.** *jointless-2* (*j-2*). **C.** *fasciated (fas).* **D.** *sun*. **E.** *yellow flesh* (r^y). **F.** *tangerine* (t^{3183}). Vertical arrows indicate insertion positions in various mutants or the break sites of the inversions in *fas* Horizontal double-headed arrows indicate the extent of deletions in mutations.

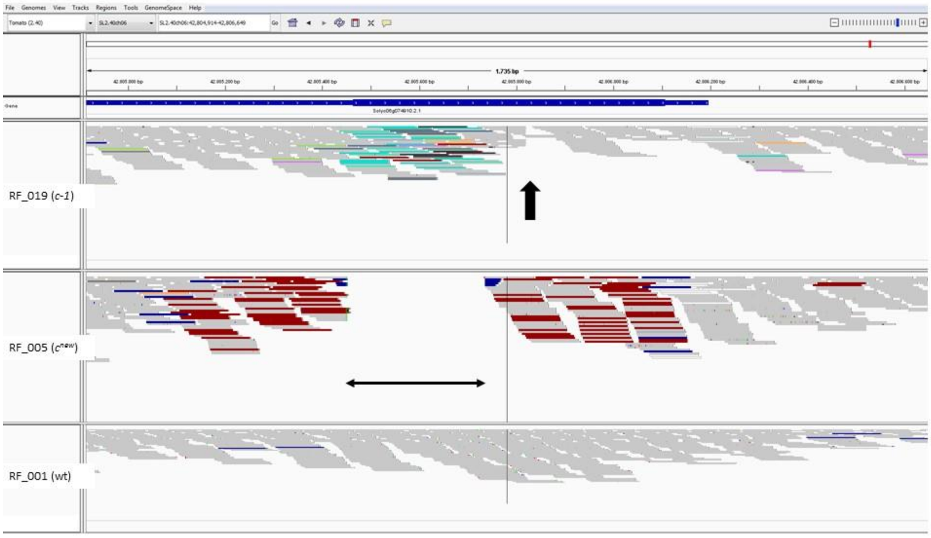

A

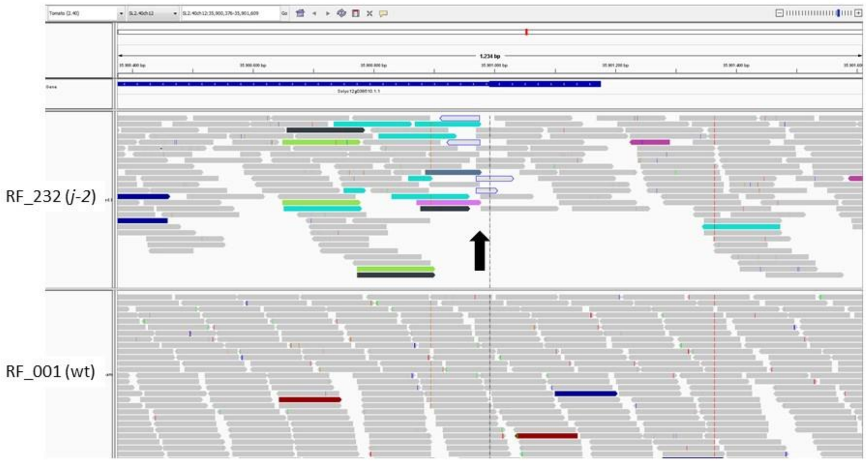

B

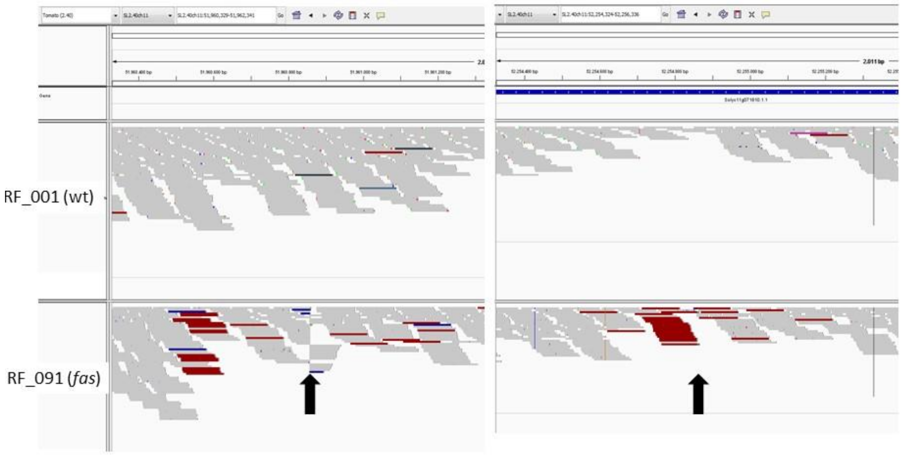

C

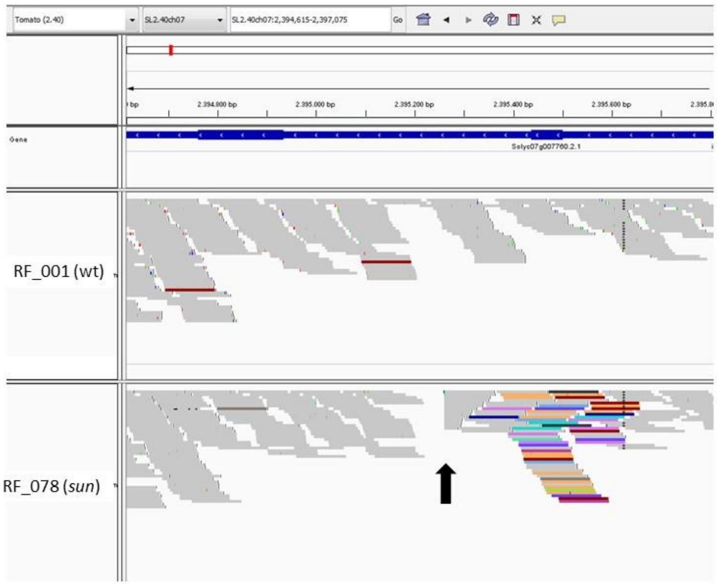
Tomato (2.40)
SL2.40ch07
SL2.40ch07:2,394,615-2,397,075
Go
Gene
Solyc07g007760.2.1
RF_001 (wt)
RF_078 (*sun*)

D

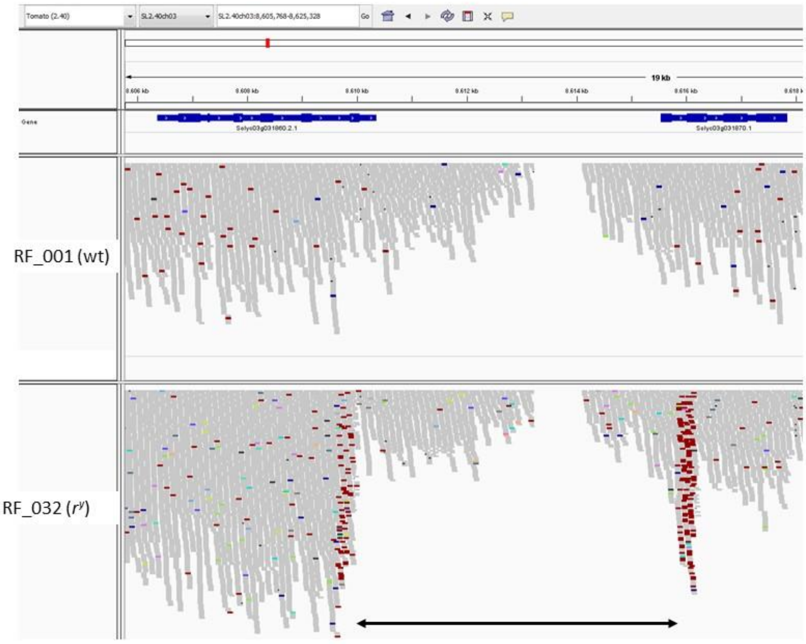
Tomato (2.40)
SL2.40ch03
SL2.40ch03:8,605,768-8,625,328
Go
19 kb
Gene
Solyc03g031860.2.1
Solyc03g031870.1
RF_001 (wt)
RF_032 (r^y)

E

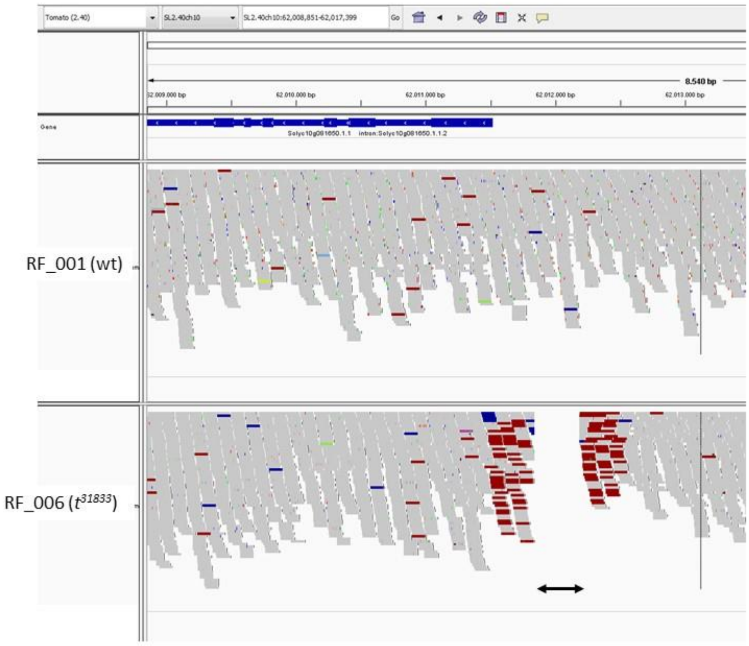

F

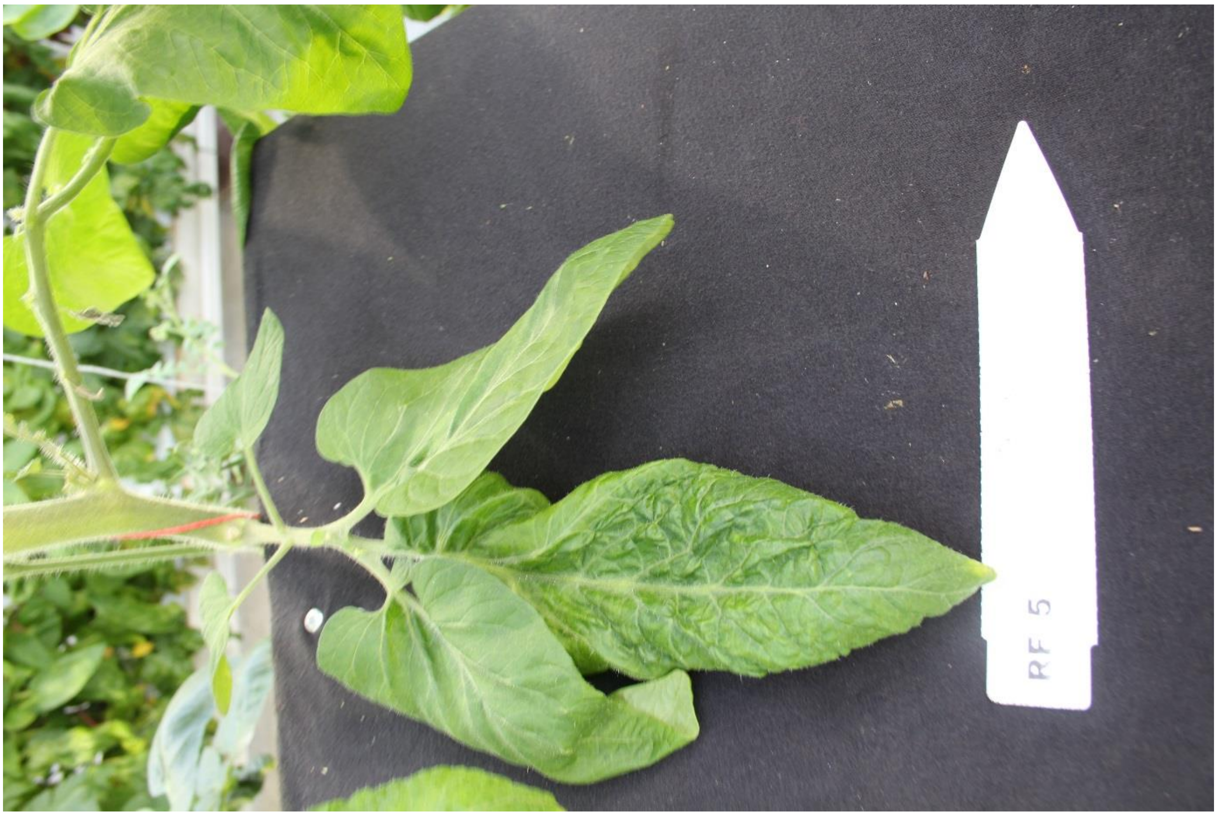

Figure S 8. *potato leaf* mutant allele (*c*) in cv. Galina (RF_005).
